# An Off‐the‐Shelf Artificial Blood Clot Hydrogel Neutralizing Multiple Proinflammatory Mediators for Pro‐Regenerative Periodontitis Treatment

**DOI:** 10.1002/advs.202504106

**Published:** 2025-05-28

**Authors:** Yini Huangfu, Zhezhe Zhao, Xiang Liu, Jingrong Wang, Yufeng Zhang, Tingting Lan, Yonglan Wang, Chenxuan Wu, Ju Zhang, Pingsheng Huang, Chuangnian Zhang, Anjie Dong, Zujian Feng, Deling Kong, Weiwei Wang

**Affiliations:** ^1^ Department of Polymer Science and Engineering Key Laboratory of Systems Bioengineering (Ministry of Education) School of Chemical Engineering and Technology Tianjin University Tianjin 300072 P. R. China; ^2^ State Key Laboratory of Advanced Medical Materials and Devices Institute of Biomedical Engineering Chinese Academy of Medical Sciences and Peking Union Medical College Tianjin 300192 P. R. China; ^3^ Department of Periodontology School and Hospital of Stomatology Tianjin Medical University Tianjin 300070 P. R. China; ^4^ College of Life Sciences Key Laboratory of Bioactive Materials (Ministry of Education) State Key Laboratory of Medicinal Chemical Biology Nankai University Tianjin 300071 P. R. China; ^5^ Research Institute of Transplant Medicine Tianjin First Central Hospital School of Medicine Nankai University Tianjin 300190 P. R. China

**Keywords:** hydrogel, inflammation modulation, periodontitis, platelet‐rich plasma, tissue regeneration

## Abstract

Periodontitis is a destructive and chronic inflammatory disease initiated and sustained by multiple proinflammatory mediators. Current therapies mainly deal with bacteria elimination, but directly addressing the over‐accumulated multiple inflammatory mediators in the periodontal microenvironment still remains a substantial challenge for regenerative periodontitis treatment. Herein, inspired by blood coagulation, an off‐the‐shelf artificial blood clot hydrogel encapsulated with platelet‐rich plasma (PRP) is reported to mitigate the deteriorative inflammatory environment in periodontitis. The hydrogel (CCS‐RSF@PRP) with a hierarchical fibers‐interwoven network structure, in which the activated platelets are enriched, is structurally similar to the native blood clot. Functionally, in addition to the function of enriching and releasing growth factors, CCS‐RSF@PRP can remarkably scavenge reactive oxygen species (ROS), neutralize endotoxin lipopolysaccharide (LPS), and proinflammatory cytokines (TNF‐α, IFN‐γ and IL‐1β), inhibit M1 macrophage polarization and induce M2 macrophage polarization, thus blocking the chronic inflammatory feedback loop in the periodontitis. In rat periodontitis model, CCS‐RSF@PRP hydrogel significantly expedits the repair of periodontium by normalizing the periodontal immune‐environment. The work highlights the importance of local immunomodulation in the treatment of periodontitis, and the engineered PRP‐derived hydrogel can mimic and expand the structure and function of native blood clot, holding great promise in treating chronic inflammatory diseases.

## Graphical abstract

CCS‐RSF@PRP hydrogel is engineered to structurally and functionally mimic the blood clot by integrating PPR, CCS, and RSF nanofibers through in situ activation of PRP. In a rat periodontitis model, CCS‐RSF@PRP hydrogel effectively promotes periodontal tissue repair, attributing to neutralizing the multiple proinflammatory mediators, thus rebuilding a pro‐reparative immune microenvironment, showing great therapeutic potential as an off‐the‐shelf artificial blood clot.

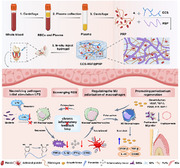

## 1 Introduction

Periodontitis is known as the “first killer” of oral health^[^ ^1^ ^]^ and is associated with systemic diseases such as diabetes^[^ ^2^ ^]^ and cardiovascular disease,^[^ ^3^ ^]^ which remains a great challenge for clinicians, generally leading to the formation of periodontal pocket, resorption of alveolar bone, and ultimately tooth loss.^[^ ^4^ ^]^ Periodontitis is a destructive chronic inflammatory disease of periodontal tissue caused by plaque microorganisms and host immune response.^[^ ^5^ ^]^ Current clinical therapies for periodontitis treatment focus on mechanical therapy and the use of antibiotics (PERIO, H_2_O_2_, chlorhexidine, and metronidazole) to combat bacterial infection, and to temporarily relieve pathogens‐associated inflammation,^[^ ^6^ ^]^ but cannot deracinate chronic inflammation and restore the structures and functions of periodontal tissues for periodontitis patients. Anti‐inflammatory drugs and cytokines have also been investigated to target the inflammation, but restricted by a difficult delivery, temporal stimulus, and unnecessary side effects.^[^ ^7^ ^]^ It is still challenging to fully address the repetitive inflammatory microenvironment to prevent further exacerbation of periodontitis and synchronously induce the regeneration of periodontium.

The pathogenic bacteria of dental plaque produce pathogen‐associated molecular patterns (PAMPs) around periodontal tissue, such as endotoxin lipopolysaccharide (LPS), which acts as an initial stimulus to induce acute inflammation response through recruitment and activation of pro‐inflammatory immune cells, including macrophages, neutrophil, and dendritic cells.^[^ ^8^ ^]^ LPS also induces the overexpression of receptor activator of nuclear factor kappa‐B (NF‐κB) ligand (RANKL) in cells, which is a key factor involved in osteoclast differentiation, leading to bone loss.^[^ ^9^ ^]^ Subsequently, the accumulation of destructive immune cells, such as macrophages, is accompanied by the production of excess reactive oxygen species (ROS) and the secretion of pro‐inflammatory chemo‐ and cytokines (interleukin‐1β (IL‐1β), interferon‐γ (INF‐γ), and tumor necrosis factor‐α (TNF‐α), et al) to amplify and sustain the inflammation through recruiting monocytes and macrophages to the lesions and polarizing them to M1 phenotype in a positive feedback loop, thus aggravating the development of chronic inflammation.^[^ ^10^ ^]^ Additionally, massive ROS can cause persistent oxidative damage to periodontal cells, and also act as cellular signaling messengers for the activation of the NF‐κB pathway and the elevation of the ratio of RANKL/OPG (osteoprotegerin), resulting in alveolar bone resorption.^[^ ^11^ ^]^ In the past decades, anti‐inflammatory drugs and cytokines, such as selective cytokine blockers for interleukin‐1 (IL‐1), interleukin‐6 (IL‐6), and TNF‐α, have been verified in the clinical treatment for a variety of acute and chronic diseases. However, a single drug or cytokine only targets a specific inflammatory pathway, which may be limited in periodontitis treatment. Hence, there is an urgent need to develop biomaterials with preponderance in effectively abolishing multiple inflammatory mediators for periodontal inflammation modulation to inhibit the progression of periodontitis and improve the regeneration of periodontal tissue.

Recently, engineered biomaterials for neutralizing inflammatory mediators have received extensive attention in improving the therapeutic outcome of inflammation‐related diseases, including sepsis, bowel disease, arthritis, and so on.^[^ ^12^ ^]^ However, most current biomaterial systems only target a specific inflammatory mediator, which may be limited in treating chronic inflammation diseases, such as periodontitis, facing a complex biological process involving multiple inflammatory mediators. It has been well recognized that platelets have versatile and identified roles in host defense and immunity, which can directly kill microbes by releasing antimicrobial peptides, and also entrap microbes and mediate aggregation of LPS and bacteria, facilitating endotoxin clearance and restricting pathogen spread.^[^ ^8^, ^13^ ^]^ Moreover, the diversity of platelet receptors such as complement receptors, Fc‐γ receptor IIa, Toll‐like receptors (TLRs), and IL‐1β receptor (IL‐1R), could govern the initial inflammatory response mediated by pro‐inflammatory chemo‐ and cytokines.^[^ ^14^ ^]^ Additionally, activated platelets will sustainedly release abundant growth factors and chemokines, which are conducive to the transformation of macrophages from pro‐inflammatory (M1) to anti‐inflammatory (M2) phenotype in the remodeling phase, promoting angiogenesis and collagen deposition.^[^ ^15^ ^]^ Thus, platelets are important to the fine equilibrium of host pro‐reparative immune and inflammatory responses. Platelet‐based biomaterials may hold great potential in immunoregulation through neutralizing inflammatory mediators, which remains to be adequately elucidated.

**Scheme 1:**
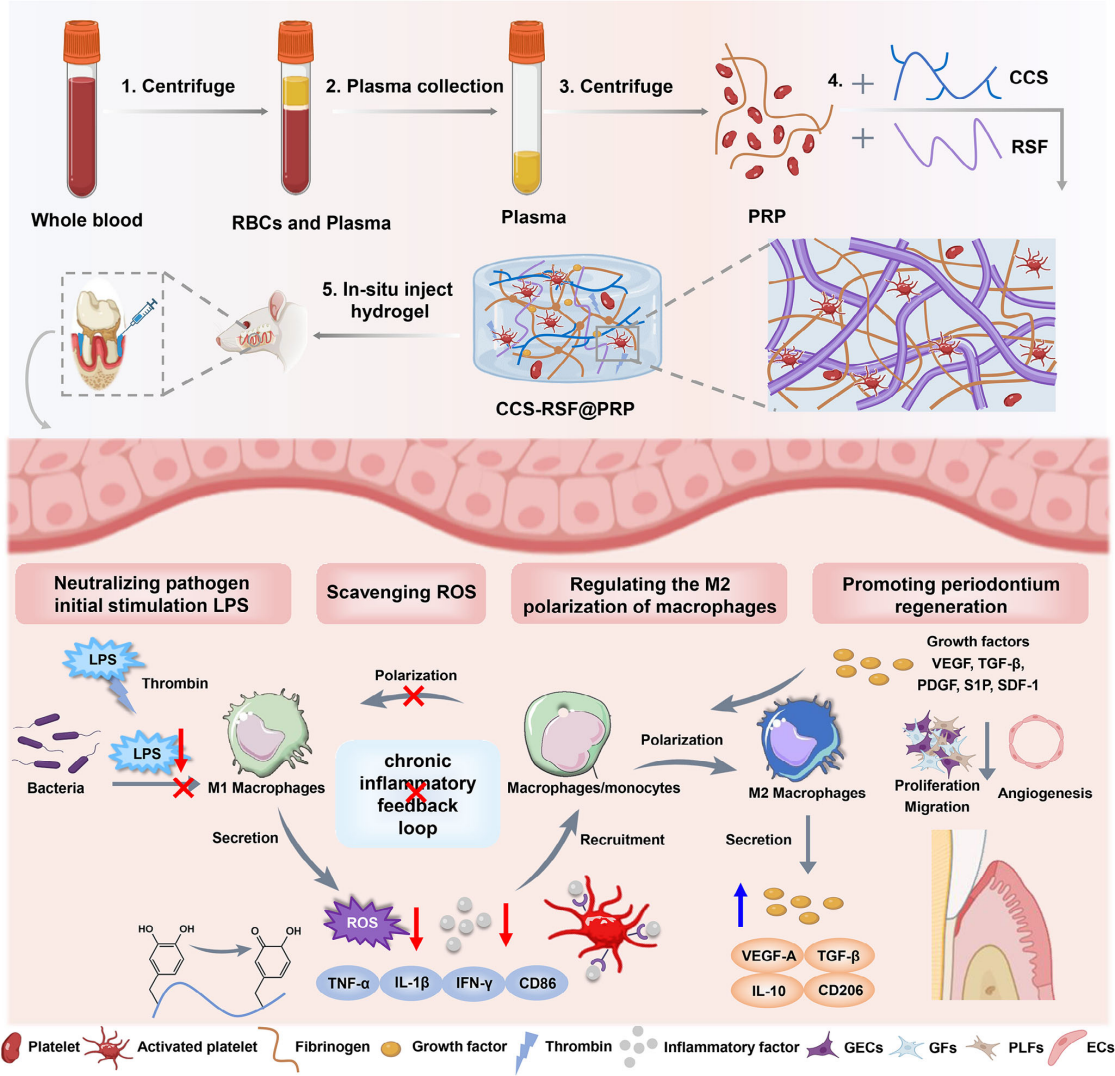
Schematic illustration of an off‐the‐shelf artificial blood clot CCS‐RSF@PRP hydrogel which could neutralize multiple proinflammatory mediators for pro‐regenerative periodontitis treatment.

Herein, inspired by the blood coagulation and the function of platelets, we elaborately engineered an off‐the‐shelf artificial blood clot hydrogel with a comprehensive superiority of neutralizing the pathogen initial stimulus LPS, scavenging ROS and eliminating proinflammatory cytokines, thus blocking the progression of chronic periodontal inflammation (**Scheme** **1**). The hybrid hydrogel was fabricated by platelet‐rich plasma (PRP), catechol‐modified chitosan (CCS), and regenerated silk fibroin (RSF) nanofibers through multiple interactions, including intermolecular interaction and in situ activation of PRP. The CCS with catechol group could act as ROS scavengers and protect cells from oxidative stress.^[^ ^16^ ^]^ And RSF nanofibers rendered the gel with a hierarchical fibers‐interwoven network structure, in which the platelets were enriched, structurally similar to the native blood clot. Moreover, CCS‐RSF hydrogel could active the autologous platelets in situ without exogenous activators to form an artificial blood clot, avoiding the risk of burst release of growth factors and potential toxicity of exogenous activators (e.g., Ca^2+^/thrombin) and providing an off‐the‐shelf product with extended shelf life. More importantly, CCS‐RSF@PRP hydrogel elicited high selectivity for capturing and binding LPS and multiple chemo‐ and cyto‐kines, including TNF‐α, IFN‐γ, and IL‐1β, and driving the macrophages polarization toward M2 phenotype, which could synergistically block the chronic inflammatory feedback loop, mitigate the destructive inflammatory microenvironment in periodontal, and restore immune homeostasis of periodontitis, owing to the diversity of platelet membrane receptors and signal factors in activated PRP. So far, developing a hydrogel that could neutralize multiple inflammatory mediators, including pathogen initial stimulus LPS, ROS, and pro‐inflammatory cytokines, remains a substantial challenge. It was experimentally verified that CCS‐RSF@PRP hydrogel showed superior therapeutic effects over commercial gel in a rat periodontitis model. CCS‐RSF@PRP hydrogel also held potentially clinical translation value for periodontitis treatment based on clinical‐approved PRP and biocompatible CCS and RSF.

## 2 Results

### 2.1 Preparation and Characterizations of CCS‐RSF@PRP Hydrogels

**Figure 1:**
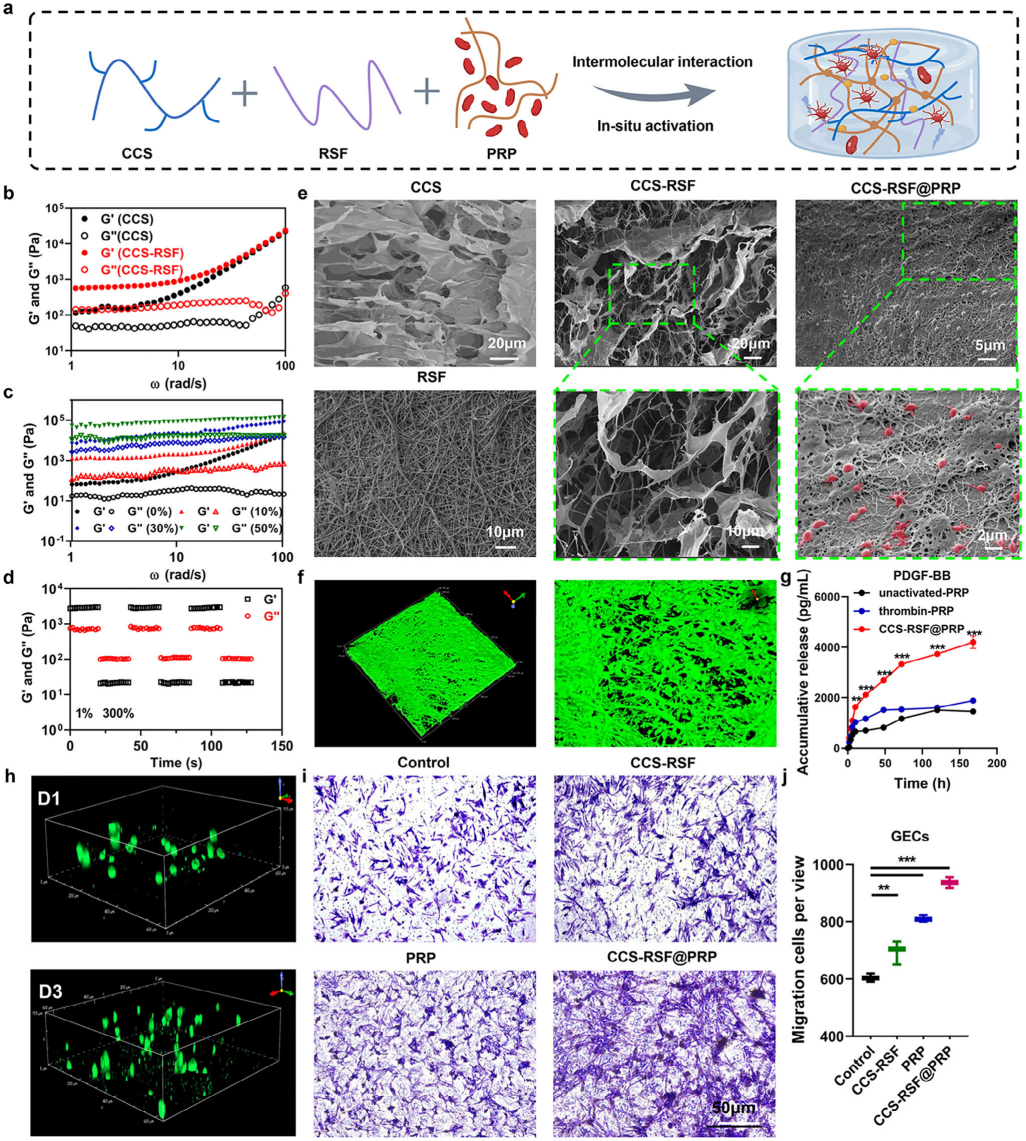
Fabrication and characterization of CCS‐RSF@PRP hydrogels. a) Schematic diagram of the fabrication of CCS‐RSF@PRP hydrogel. Rheological analysis of CCS‐RSF@PRP hydrogels with different concentrations of b) RSF nanofibers and c) PRP solution (v/v). d) The self‐healing analysis of CCS‐RSF@PRP hydrogel. e) Representative SEM images of lyophilized CCS hydrogel, RSF nanofibers, CCS‐RSF, and CCS‐RSF@PRP hydrogel. f) The 3D construction of CLSM images of CCS‐RSF hydrogel. g) The accumulative release of PDGF‐BB from unactivated PRP, thrombin‐activated‐PRP, and CCS‐RSF@PRP hydrogel (n = 3). h) The 3D construction of CLSM images of GECs after incubation within hydrogel on day 1 and day 3 based on the z‐axis stack. Viable cells were stained with calcein‐AM. i, j) Representative images and quantitative analysis of Transwell migration assay for GECs (n = 3).

Accordingly, CCS molecules were first synthesized by conjugating hydrocaffeate (HCA) to chitosan using standard EDC‐NHS chemistry (Figure S1a, Supporting Information). And the successful synthesis of CCS was confirmed by ^1^H NMR and UV‐vis spectra (Figure S2a‐c, Supporting Information). The conjugation ratio of HCA to chitosan molecules was calculated at ≈11.8%. Then, RSF nanofibers were obtained through an electrostatic spinning process and fragmented in a cryogenic milling chamber filled with liquid nitrogen (Figure S1b, Supporting Information). The CCS‐RSF hydrogel was prepared through intermolecular interaction between CCS and RSF nanofibers, including hydrogen bonds and electrostatic interactions. The composition of CCS‐RSF hydrogel was characterized by FTIR spectra (Figure S3, Supporting Information). The characteristic peaks at 760, 1400^,^ and 1730 cm^−1^ of CCS‐RSF hydrogel were attributed to C─H bending vibration peak on the ortho‐substituted aromatic ring, C─N stretching vibration peak in CCS, and C═O stretching vibration peak in RSF, respectively. Finally, CCS‐RSF@PRP hydrogel was obtained by mixing PRP solution with CCS‐RSF hydrogel and incubation at 37 °C to adequately active PRP (Scheme 1, **Figures** **1**a and Figure S4, Supporting Information).

The mechanical properties of hydrogels were measured by dynamic rheology analysis to optimize the formulation of hydrogels. As shown in Figure 1b, the values of G′ (storage modulus) were greater than that of G″ (loss modulus) for CCS and CCS‐RSF hydrogel, indicating the formation of a solid‐like hydrogel with elastic dominant properties. With the increase of RSF concentration, the values of G′ and G″ increased gradually (Figure S5a, Supporting Information), owing to the molecular interactions and particle enhancement of RSF. It's worth noting that excessive RSF nanofibers (more than 10 mg mL^−1^) would result in phase separation, compromising the stability of hydrogels. Compared to CCS‐RSF hydrogel, CCS‐RSF@PRP hydrogel showed an order of magnitude increase in G′ and G″ values (Figure 1c; Figure S5b, Supporting Information), suggesting abundant fibrinogen in the PRP was converted into tightly packed fibrin network owing to in situ activation, spontaneously penetrating into and entangling with CCS‐RSF hydrogel network. Further, hydrogels were subjected to strain sweep to figure out the gel‐sol transition behavior. Results showed that CCS‐RSF@PRP hydrogel displayed the typical non‐Newtonian shear thinning (pseudoplastic) behavior with gel‐to‐sol phase transition at a strain of ≈115%, suggesting the injectability of CCS‐RSF@PRP hydrogel (Figure S5c, Supporting Information). To evaluate the self‐healing ability, step‐strain measurement was carried out. As shown in Figure 1d, both G′ and G″ modulus dropped sharply upon applying a large‐amplitude oscillating strain (300%), and quickly recovered at low shear strain (1%), and this process was recyclable owing to reversible hydrogen bonds and electrostatic interaction. Additionally, time‐sweep rheological tests revealed a rapid gelation time of ≈2.3 min at a physiological temperature (37 °C) (Figure S6a, Supporting Information), ensuring convenient clinical preparation. Meanwhile, we conducted a rheological analysis to quantify the temperature (4‐50 °C)‐dependent behavior of CCS‐RSF@PRP hydrogel (Figure S6b, Supporting Information). The results demonstrated that the hydrogel maintained stable gel state within the clinically relevant temperature range of 4–47 °C, where the storage modulus (G′) consistently exceeded the loss modulus (G″). Above 47 °C, the gradual decrease in G′ and G″ lead to degelling (G′ < G″), due to the thermal damage of PRP fiber network. Furthermore, the swelling ratio of CCS‐RSF@PRP hydrogel increased after immersion in PBS or artificial saliva solution under dynamic conditions on a shaker, mimicking the dynamic factors like salivary and masticatory forces in oral environment, and reached an equilibrium at 8 h and 2 h, respectively (Figure S7a,b, Supporting Information). The swelling ratio of CCS‐RSF@PRP hydrogel then decreased due to degradation, particularly in artificial saliva solution. In addition, the degradation behavior of CCS‐RSF@PRP hydrogel in both PBS and artificial saliva under dynamic conditions confirmed a gradual mass loss in artificial saliva ≈65% over 7 days (Figure S8a,b, Supporting Information). These results demonstrated that injectable and self‐healing CCS‐RSF@PRP hydrogel was successfully fabricated, which could be used as a physical filler for the treatment of periodontitis.

The internal structures of CCS hydrogel, RSF nanofibers, CCS‐RSF, and CCS‐RSF@PRP hydrogels were investigated by scanning electron microscopy (SEM). As shown in Figure 1e, a typical 3D sponge‐like microstructure was observed in CCS hydrogel, and a homogeneous nanofiber structure with an average diameter of 0.6 µm was obtained in RSF electrospinning membrane (Figure S9, Supporting Information). Interestingly, SEM and confocal laser scanning microscopy (CLSM) images of CCS‐RSF hydrogel revealed that these nanofibers were connected to CCS hydrogel network due to intermolecular interaction between CCS and RSF (Figure 1e,f), exhibiting a hierarchical fibers‐interwoven network microarchitecture similar to the native blood clot,^[^ ^17^ ^]^ which was expected to benefit cell proliferation and tissue remodeling. Notably, numerous pseudopods were observed for platelets presented in CCS‐RSF@PRP hydrogel, indicating PRP was activated in situ by CCS‐RSF hydrogel without exogenous stimulus.

Healing‐related cell proliferation and migration are indispensable processes during tissue regeneration and play an important role in tissue fusion.^[^ ^18^ ^]^ Artificial blood clot scaffolds could provide functions, structural integration, and suitable growth conditions for cells, which is beneficial to tissue repair and regeneration.^[^ ^17^, ^19^ ^]^ Moreover, activated PRP would release a large number of growth factors and chemokines (e.g., platelet‐derived growth factor‐BB (PDGF‐BB), transforming growth factor‐beta (TGF‐β), vascular endothelial growth factor (VEGF), fibroblast growth factor (FGF), and stromal cell‐derived factor‐1 (SDF‐1)), which are able to promote cell proliferation and migration.^[^ ^15^, ^20^ ^]^ Herein, PDGF‐BB, TGF‐β, and VEGF were selected as typical growth factors to investigate the activation of PRP by CCS‐RSF hydrogel. Compared with unactivated PRP and thrombin‐activated PRP, CCS‐RSF@PRP hydrogel presented a significantly higher efficiency in PDGF‐BB, TGF‐β1, and VEGF production with a sustained release profile (Figure 1g; Figure S10a,b, Supporting Information). Especially, the amount of PDGF‐BB released from PRP was an order of magnitude more than that of TGF‐β1 and VEGF. Moreover, the secretions of ATP and calcium from activated PRP induced by thrombin and CCS‐RSF@PRP were also measured to verify the activation of PRP. The level of ATP and Ca^2+^ in all groups were increased after activation compared with unactivated PRP. Moreover, ATP and Ca^2+^ secretions in CCS‐RSF@PRP group were significantly elevated and statistically higher than that of thrombin‐PRP (Figure S11a,b, Supporting Information). These results revealed that CCS‐RSF@PRP could effectively activate PRP in situ and sustainedly release multiple growth factors including PDGF‐BB, TGF‐β1 and VEGF.

Subsequently, the influence of CCS‐RSF@PRP hydrogel on cell proliferation was explored by CCK‐8 assay. The results demonstrated that CCS‐RSF@PRP hydrogel exhibited good cytocompatibility on gingival epithelial cells (GECs), gingival fibroblasts (GFs), periodontal ligament fibroblasts cells (PLFs) and cementoblasts (OCCM‐30). Additionally, owing to the sustained release of multiple growth factors, CCS‐RSF@PRP hydrogel facilitated periodontal associated‐cells proliferation during the 7‐day culture time (Figure S13a–d, Supporting Information). Live/dead assay (Figure S14a,b, Supporting Information) indicated that after culturing with CCS‐RSF@PRP hydrogel for 1 day, a large number of survival cells (green fluorescence) and almost no dead cells (red fluorescence) were detected. The amount of survival cells was increased in CCS‐RSF@PRP hydrogel group compared with other groups, due to in situ activation of PRP and subsequent proliferation‐related factors release. To verify the fibrous network of CCS‐RSF@PRP hydrogel on cell proliferation, GECs were seeded within the hydrogel. As shown in Figure 1h, GECs were visualized by green fluorescence dots, and continually proliferated in the 3D hydrogel bulk as the incubation time prolonged, suggesting that artificial blood clot hydrogels with hierarchical fibers‐structure could provide a favorable environment for cell proliferation. The tube formation assay was carried out for the assessment of angiogenesis in vitro. After 6‐h incubation, CCS‐RSF@PRP hydrogel significantly promoted the tubular formation with a higher junction number compared to other groups, owing to the sustained release of PDGF‐BB and VEGF from activated PRP (Figure S15a, b, Supporting Information). Moreover, the cell migration of GECs, GFs, and human umbilical vein endothelial cells (HUVECs) was assessed by Transwell migration assay (Figure 1i,j; Figures S16a,b and S17a,b, Supporting Information). The cell migration of GECs, GFs, and HUVECs cultured with CCS‐RSF@PRP hydrogel was significantly enhanced in comparison to other groups. These results indicated that CCS‐RSF@PRP hydrogels with blood clot‐mimetic structure and sustained release of growth factors could not only facilitate cell proliferation but also promote vessel formation and cell migration in vitro, benefiting the regeneration and angiogenesis of impaired tissues.

### 2.2 Neutralization of Multiple Inflammatory Mediators by CCS‐RSF@PRP Hydrogels

**Figure 2:**
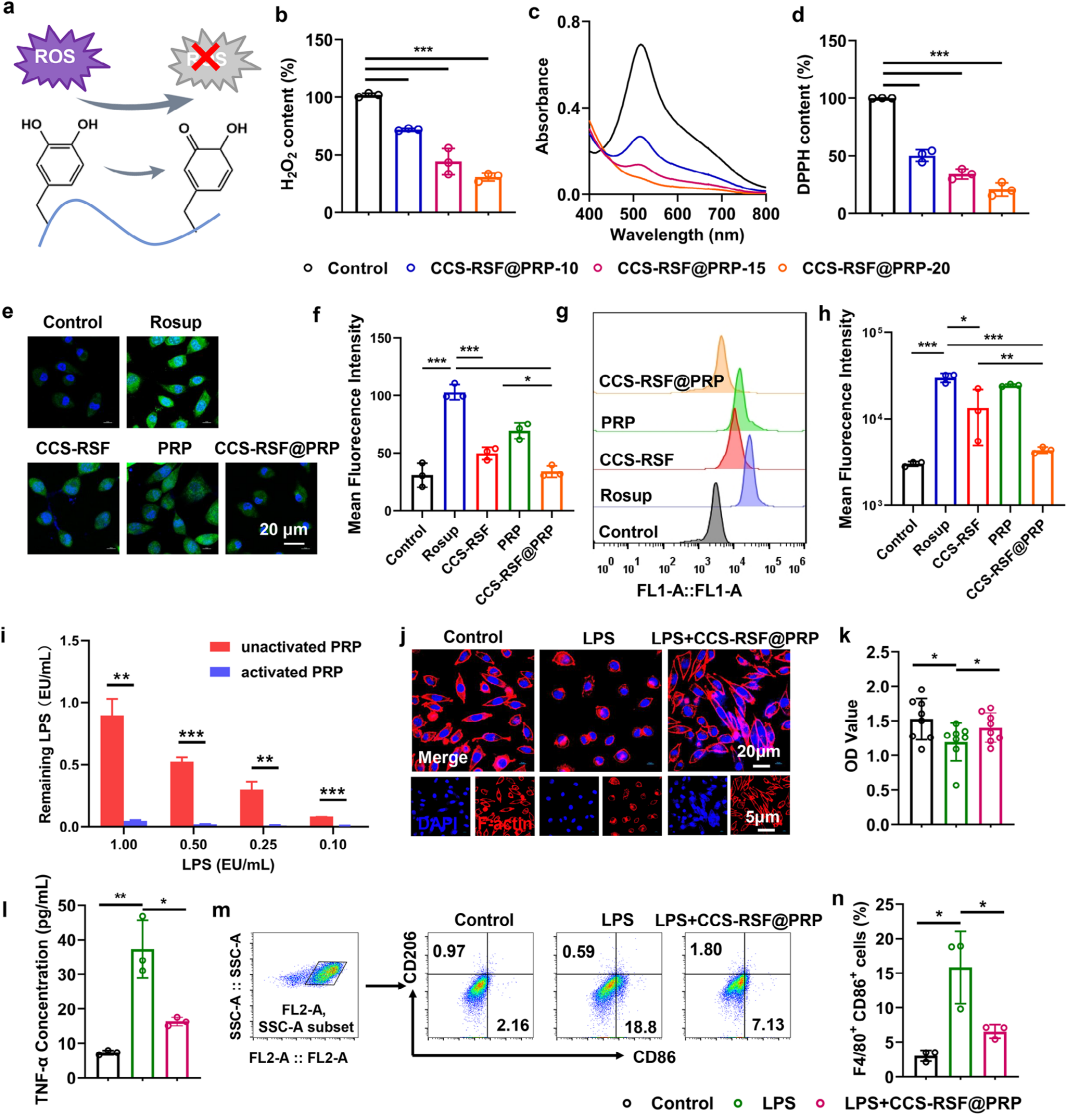
CCS‐RSF@PRP hydrogels scavenged inflammatory mediators of ROS and LPS. a) The ROS scavenging mechanism of CCS‐RSF@PRP hydrogel. b) H_2_O_2_ scavenging efficiency of CCS‐RSF@PRP hydrogel with CCS concentration of 10, 15, and 20 mg mL^−1^, respectively (n = 3). c) UV‐vis spectra of DPPH after incubation with CCS‐RSF@PRP hydrogels. d) DPPH scavenging capabilities of CCS‐RSF@PRP hydrogels (n = 3). e) Representative images of DCFH‐labeled GECs after receiving different treatments. f) Quantitative analysis of mean fluorescence intensity of DCFH (n = 3). Histogram of flow cytometry (g) and corresponding mean fluorescence intensity (h) of GECs after receiving different treatments (n = 3). i) Quantification of LPS neutralization by activated or unactivated PRP as a function of LPS amount (n = 3). j) Representative CLSM images of GECs treated by LPS and LPS with CCS‐RSF@PRP hydrogel (red: F‐actin; blue: cell nuclear). k) Cell viability of GECs cultured with LPS and LPS with CCS‐RSF@PRP hydrogel evaluated by CCK‐8 assay (n = 8). l) Concentration of inflammatory factors TNF‐α in the cell supernatant of GECs (n = 3). m) Flow cytometry analysis of CD206 and CD86 expression (gated on F4/80^+^ cells). n) Percentage of M1 (F4/80^+^CD86^+^) type macrophages (n = 3).

Accumulation of excessive ROS is a typical feature of periodontitis tissues caused by bacteria infection and obstructs the regeneration of periodontal tissues. In this study, the catechol group was introduced in CCS‐RSF@PRP hydrogel, which is widely used as an antioxidant molecule that can be oxidized to quinones to trap and reduce free radicals including O_2_^‐^, ·OH and H_2_O_2_,^[^ ^21^ ^]^ and protect cells from oxidative stress^[^ ^22^ ^]^ (**Figure** **2**a). To characterize the antioxidant properties of CCS‐RSF@PRP hydrogel, we evaluated the scavenging ability of CCS‐RSF@PRP hydrogel on superoxide anion, hydroxyl free radical, and H_2_O_2_. Compared with the control group, CCS‐RSF@PRP hydrogel could effectively eliminate superoxide anion (Figure S18a,b, Supporting Information), hydroxyl free radical anion (Figure S18c,d, Supporting Information) and H_2_O_2_ (Figure 2b). The scavenging rate of these free radicals by CCS‐RSF@PRP hydrogel showed a CCS content‐dependent behavior. Furthermore, a 1,1‐diphenyl‐2‐picrylhydrazyl (DPPH) free radical scavenging assay was also conducted to investigate the antioxidative properties of CCS‐RSF@PRP hydrogel. UV‐vis spectra showed that the DPPH peak at 517 nm was dropped after incubation with CCS‐RSF@PRP hydrogel (Figure 2c). The quantitative analysis of the remaining DPPH in CCS‐RSF@PRP‐treated DPPH solution showed that the DPPH‐scavenging capability of the hydrogel depended on CCS concentration (Figure 2d).

To test the cellular ROS scavenging activity of CCS‐RSF@PRP hydrogel, GECs were pretreated with Rosup reagent, and then fresh media containing CCS‐RSF hydrogel, PRP, and CCS‐RSF@PRP hydrogel were added. DCFH‐DA was used as a ROS probe, which could be oxidized and turned into DCF with green fluorescence. As shown in Figure 2e,f, compared with the Rosup group, GECs treated with CCS‐RSF@PRP hydrogel exhibited significantly weaker green fluorescence intensity, indicating a lower level of intracellular ROS. The level of intracellular ROS was further quantitively characterized by flow cytometry (Figure 2g,h). The mean fluorescence intensity of cells treated with CCS‐RSF@PRP hydrogel was significantly reduced compared with Rosup‐treated cells. It was demonstrated that CCS‐RSF@PRP hydrogel was more effective in reducing intracellular ROS compared with CCS‐RSF hydrogel and PRP, which may benefit the immunomodulation activity of CCS‐RSF@PRP hydrogel. Moreover, we explored the effects of H_2_O_2_ on the cell viability of GECs (Figure S19a, Supporting Information). Compared with a control group, H_2_O_2_ would cause oxidative stress damage and refrain the cell viability. Owing to the ROS scavenging capability, cells treated with CCS‐RSF@PRP hydrogel could maintain a high cell viability. In addition, flow cytometry analysis was further performed on apoptotic cells using Annexin V‐FITC apoptosis detection kit (Figure S19b,c, Supporting Information). Compared with control group, 57.4% of GECs were apoptotic after H_2_O_2_ stimulation, with 47.4% being early apoptotic. However, CCS‐RSF@PRP hydrogel treatment greatly reduced the percentage of apoptotic cells to 22.13%, owing to the ROS scavenging capability of catechol groups in CCS‐RSF@PRP. These results indicated that CCS‐RSF@PRP hydrogel could scavenge multiple ROS, attributing to the introduction of catechol group, thus reducing oxidative stress damage to periodontal cells in the inflammatory environment of periodontitis.

The pathogenic bacteria of dental plaque would release endotoxin LPS, which initiates and sustains the chronic inflammation through excessive activation of toll‐like receptors (TLRs). Therefore, we further explored the neutralizing effect of CCS‐RSF@PRP hydrogel on LPS. As shown in Figure 2i, compared with unactivated PRP, CCS‐RSF@PRP hydrogel‐activated PRP could potently neutralize LPS due to release of thrombin from the activated platelet, which has been demonstrated to exert anti‐endotoxic functions in vitro and in vivo.^[^ ^8^, ^23^ ^]^ Moreover, to further demonstrate the influence of LPS on cell behavior and the neutralization capability of CCS‐RSF@PRP, GECs were cultured in a medium containing LPS with or without CCS‐RSF@PRP. As shown in Figure 2j, the change in cell morphology was verified by labeling the F‐actin and cell nuclear with Alexa Fluor 555‐conjugated phalloidine (red) and DAPI (blue), respectively. The CLSM images of GECs demonstrated that the cell morphology was nearly spherical shape under LPS stimulation, indicating that LPS could cause inflammation and change the cytoskeleton of GECs, while the cell morphology remained normal spindle shape after being treated with CCS‐RSF@PRP hydrogel. Moreover, as shown in Figure 2k,l, LPS treatment could inhibit cell viability and markedly elevate the expression level of inflammatory factor TNF‐α. However, the effective binding of LPS by CCS‐RSF@PRP hydrogel revealed a shielding effect on the inhibition of cell viability and downregulated the expression of TNF‐α. To better mimic the multifactorial stimulation in physiological periodontitis, LPS+H_2_O_2_ co‐treatment were also conducted to evaluate the therapeutic outcomes of CCS‐RSF@PRP hydrogel. Under LPS+H_2_O_2_ stimulation, GECs exhibited significant morphological alterations, transitioning from the typical spindle shape to an abnormal spherical morphology (Figure S20a, Supporting Information), accompanied by a marked reduction in cell viability (Figure S20b, Supporting Information). In contrast, GECs treated with CCS‐RSF@PRP hydrogel under identical LPS+ROS conditions retained near‐normal spindle morphology and exhibited restored cell viability, confirming the dual capacity of CCS‐RSF@PRP hydrogel to scavenge both ROS and LPS.

Since LPS is a typical stimulant to induce the proinflammatory (M1) phenotype macrophage polarization, it plays an important role in initiating and sustaining the inflammatory immune response. Flow cytometry was employed to quantificationally verify the polarization of bone marrow‐derived macrophages (BMDMs). The results in Figure 2m and Figure S21a (Supporting Information) showed that the expression level of CD86 (a typical marker for M1‐type macrophages) was significantly elevated after LPS treatment, confirming its proinflammatory activity. After co‐culture with CCS‐RSF@PRP hydrogel, the expression level of CD86 was obviously downregulated, suggesting that CCS‐RSF@PRP hydrogel could eliminate the pro‐inflammatory activity of LPS through neutralization. The percentage of M1 type macrophages determined by flow cytometry (Figure 2n; Figure S21b, Supporting Information) also confirmed that the M1 type (F4/80^+^CD86^+^) macrophages were significantly reduced after CCS‐RSF@PRP treatment. Furthermore, we established a coculture system to investigate the crosstalk between macrophages and GECs. As shown in Figure S22a (Supporting Information), macrophages derived from BMDMs were seeded in the bottom chamber and were subsequently stimulated by PBS, LPS alone, or LPS in addition with hydrogels. After incubation for 2 days, GECs were further seeded on the Transwell insert chambers. It was observed that LPS typically induced the M1‐type macrophage polarization, thus inhibiting the downward migration of GECs by secreting inflammatory factors^[^ ^24^ ^]^ (Figure S22b,c, Supporting Information). The number of migrating cells was significantly improved in CCS‐RSF@PRP group compared with that of other groups, owing to efficient LPS neutralization of CCS‐RSF@PRP hydrogel, which inhibited M1 type macrophage polarization and whittled inflammation response (Figure S21a,b, Supporting Information). The sustained release of multiple growth factors from in situ activated PRP could also contribute to cell migration. In addition, TRAP (a specific marker enzyme of osteoclasts) activity assay of osteoclasts was measured (Figure S23, Supporting Information). Compared with RANKL group (positive control), RAW cells treated with CCS‐RSF@PRP hydrogel exhibited inferior TRAP activity, indicating alleviative bone resorption due to the immunoregulatory function of CCS‐RSF@PRP in remitting deteriorative inflammation environment. Overall, these results indicated that CCS‐RSF@PRP hydrogels hold a high detoxification capacity against ROS and LPS, abolished the macrophage M1‐phenotype polarization to further improve cell migration in the inflammatory environment and inhibited bone resorption, which potentially devoted to mitigate the chronic inflammation and promote periodontium repair in periodontitis treatment.

**Figure 3:**
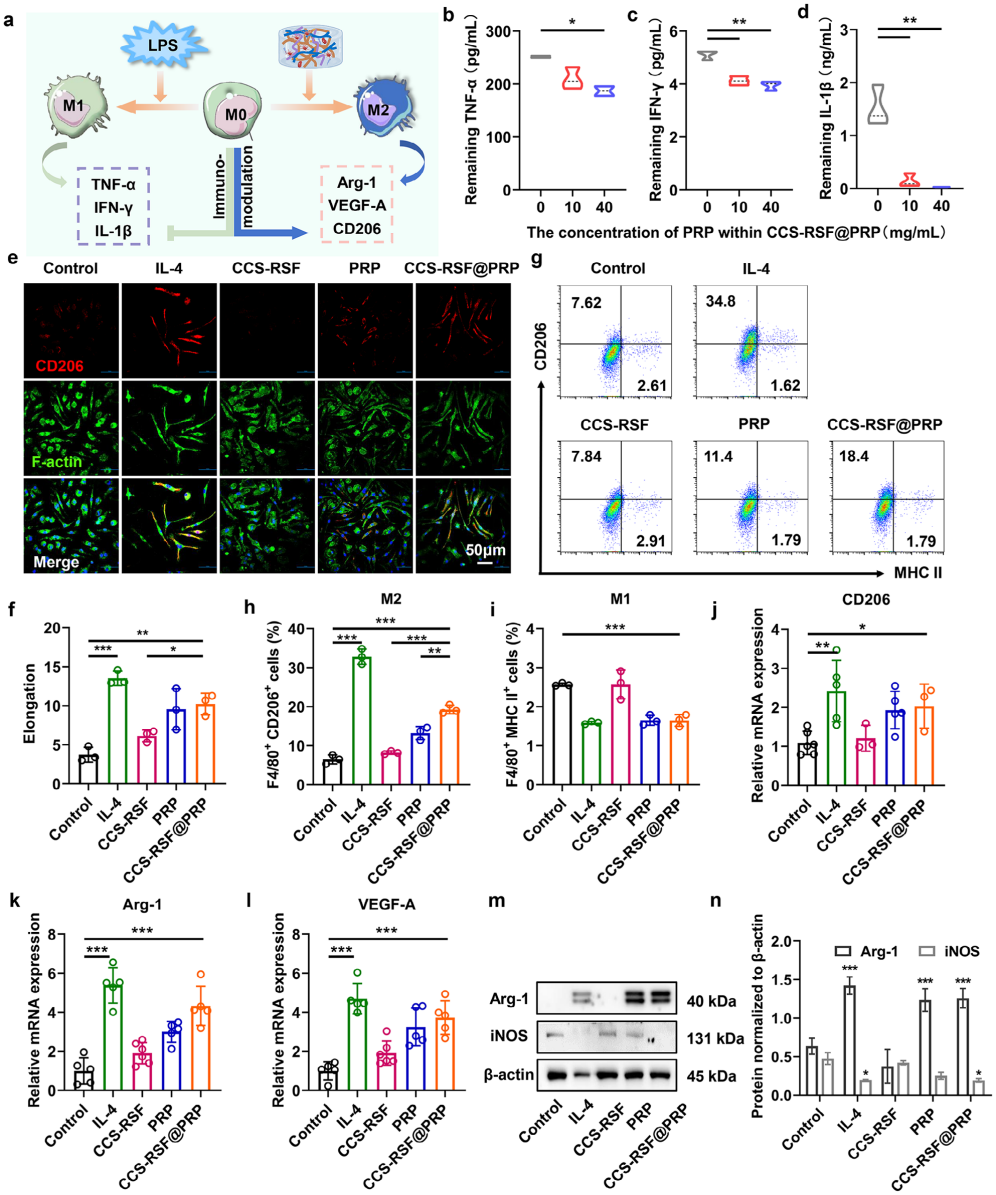
CCS‐RSF@PRP hydrogels neutralized proinflammatory cytokines and harnessed macrophages polarization toward M2 phenotype. a) Schematic diagram of macrophages polarization toward M2 phenotype induced by CCS‐RSF@PRP hydrogels. Neutralization of proinflammatory cytokines, including b) TNF‐α, c) IFN‐γ, and d) IL‐1β by CCS‐RSF@PRP hydrogel (n = 3). e) Representative CLSM images of BMDMs treated by IL‐4, CCS‐RSF, PRP, or CCS‐RSF@PRP hydrogel for 48 h, (red: CD206; green: F‐actin; blue: cell nuclear). f) The elongation of BMDMs (n = 3). g) Flow cytometry analysis of CD206 and MHC II expression (gated on F4/80^+^ cells). Percentage of h) M2 (F4/80^+^CD206^+^) and i) M1 (F4/80^+^MHC II^+^) type macrophages (n = 3). Relative mRNA expression of j) CD206, k) Arg‐1, and l) VEGF‐A (n = 5). m, n) Protein expression level of Arg‐1 and iNOS in treated macrophages determined by western blot (n = 3).

Furtherly, diverse platelet receptors, such as CD40, CD121b, and CD62P, have been reported to interact with pro‐inflammatory factors, including TNF‐α, IL‐1β and IFN‐γ.^[^ ^25^ ^]^ The protein expression level of CD62P (Figure S24, Supporting Information), a marker of platelet activation, was significantly higher in the activated platelet group, in comparison to the unactivated platelet group. Meanwhile, CD40 and CD121b were also expressed in platelet activated by CCS‐RSF. These results demonstrated that the specific family of receptors in platelets in CCS‐RSF@PRP could be activated, which may participate in targeted binding with pro‐inflammatory factors. Hence, the neutralization of proinflammatory cytokines, including TNF‐α, IFN‐γ and IL‐1β, by activated PRP was assessed. Cytokines were pre‐incubated with activated PRP, and the cytokine concentrations in the supernatant were then quantified by ELISA. As shown in **Figure** **3**a–d, with the concentration of activated PRP increased, the remaining amount of proinflammatory cytokines in the supernatant including TNF‐α, IFN‐γ, and IL‐1β was significantly decreased, suggesting the effective neutralization ability of the activated platelet toward these cytokines, then blocking their downstream signaling activation. Macrophages are important immune cells, closely involved in immune homeostasis for tissue reconstruction through secreting inflammation‐related chemo‐ and cytokines. Additionally, macrophages are highly heterogeneous and plastic, which could be classically divided into proinflammatory M1 and anti‐inflammatory M2 phenotype macrophages. It was reported that the platelets membrane contain sphingosine1‐phosphate (S1P) and phosphatidylserine, respectively, which can act as “find me” and “eat me” signaling molecules to recruit and polarize M2 macrophages in efferocytosis.^[^ ^26^ ^]^ Moreover, PRP is rich in a variety of growth factors and chemokines that could contribute to endogenous cells recruitment and the inhibition of inflammatory response (Figure 3a). Thus, the macrophage polarization effect of CCS‐RSF@PRP hydrogels was characterized, and IL‐4 was used as a positive control. First, to study the changes in cell morphology after treatment, FITC‐modified phalloidin was used to label the F‐actin, and the characteristic surface biomarker CD206 was used to identify M2‐type macrophages. As shown in Figure 3e, in comparison with the control group, BMDMs incubated with IL‐4 and CCS‐RSF@PRP hydrogel both showed stronger red fluorescence intensity of CD206 staining, indicating that macrophages were polarized into M2‐type phenotype. Compared with CCS‐RSF hydrogel and PRP groups, obvious pseudopod‐like structure and prominent elongation of macrophages in CCS‐RSF@PRP hydrogel group also suggested that CCS‐RSF@PRP hydrogel could effectively prime macrophages toward M2 phenotype (Figure 3f). Flow cytometry (Figure 3g–i) demonstrated that the percentage of M2 type (F4/80^+^CD206^+^) macrophages treated by CCS‐RSF@PRP hydrogel was 18.4%, which was significantly higher than that in control (7.62%), CCS‐RSF hydrogel (7.84%) and PRP (11.4%) group, while that of M1 type (F4/80^+^MHC II^+^) macrophages maintained at a low level. Considering that TGF‐β released from PRP is a multifunctional cytokine with important biological effect in regulating inflammatory processes, we also conducted experiments on macrophages polarization with the addition of TGF‐β as a positive control (Figure S25a,b, Supporting Information). TGF‐β significantly increased the proportion of M2 (F4/80^+^CD206^+^) macrophages (10.1%) compared with control group (0.94%), while the percentage of M2 type macrophages in IL‐4 group (34.4%) and CCS‐RSF@PRP group (24.0%) was significantly higher than that in control or TGF‐β group. These data demonstrated the release of anti‐inflammatory cytokines and growth factors from CCS‐RSF@PRP hydrogel could contribute to the regulation of macrophage polarization. Furtherly, RT‐qPCR was employed to identify the relative mRNA expression level of macrophages after being treated with CCS‐RSF@PRP hydrogel. Results (Figure 3j–l) demonstrated that the mRNA expression level of CD206, Arg‐1, and VEGF‐A related to M2 macrophages in CCS‐RSF@PRP hydrogel group were significantly elevated. The expression of related proteins including Arg‐1 and iNOS of macrophages after being treated with CCS‐RSF@PRP hydrogel were investigated by western blot. As shown in Figure 3m,n, the protein expression level of Arg‐1 (M2 marker) was higher in CCS‐RSF@PRP hydrogel group than in the control group, and downregulation of iNOS expression (M1 marker) was also observed. Therefore, CCS‐RSF@PRP hydrogel could effectively mediate the macrophage polarization toward M2 type and meanwhile did not cause the expression of inflammation‐related factors.

To further investigate the repolarization effect of CCS‐RSF@PRP hydrogel on M1 macrophages, macrophages were pre‐incubated with LPS. Then, the obtained M1 macrophages were treated with PBS, IL‐4 or CCS‐RSF@PRP hydrogel. Flow cytometry analysis (Figure S26a–c, Supporting Information) revealed that upon CCS‐RSF@PRP hydrogel treatment, the ratio of M2 type (F4/80^+^CD206^+^) macrophages was elevated compared with that in the control group, while the M1 type (F4/80^+^MHC II^+^) macrophages were remarkably decreased. Simultaneously, the ratio of M2/M1 was increased in CCS‐RSF@PRP hydrogel group, consistent with the IL‐4 positive group (Figure S26d, Supporting Information). Together, these results indicated the superiority of CCS‐RSF@PRP hydrogel in mediating the immune homeostasis by driving the macrophage polarization toward M2 phenotype, which may further help to eliminate the proinflammatory mediators and suppress the inflammatory response during tissue repair.

### 2.3 In Vivo Periodontitis Treatment by CCS‐RSF@PRP Hydrogels

**Figure 4:**
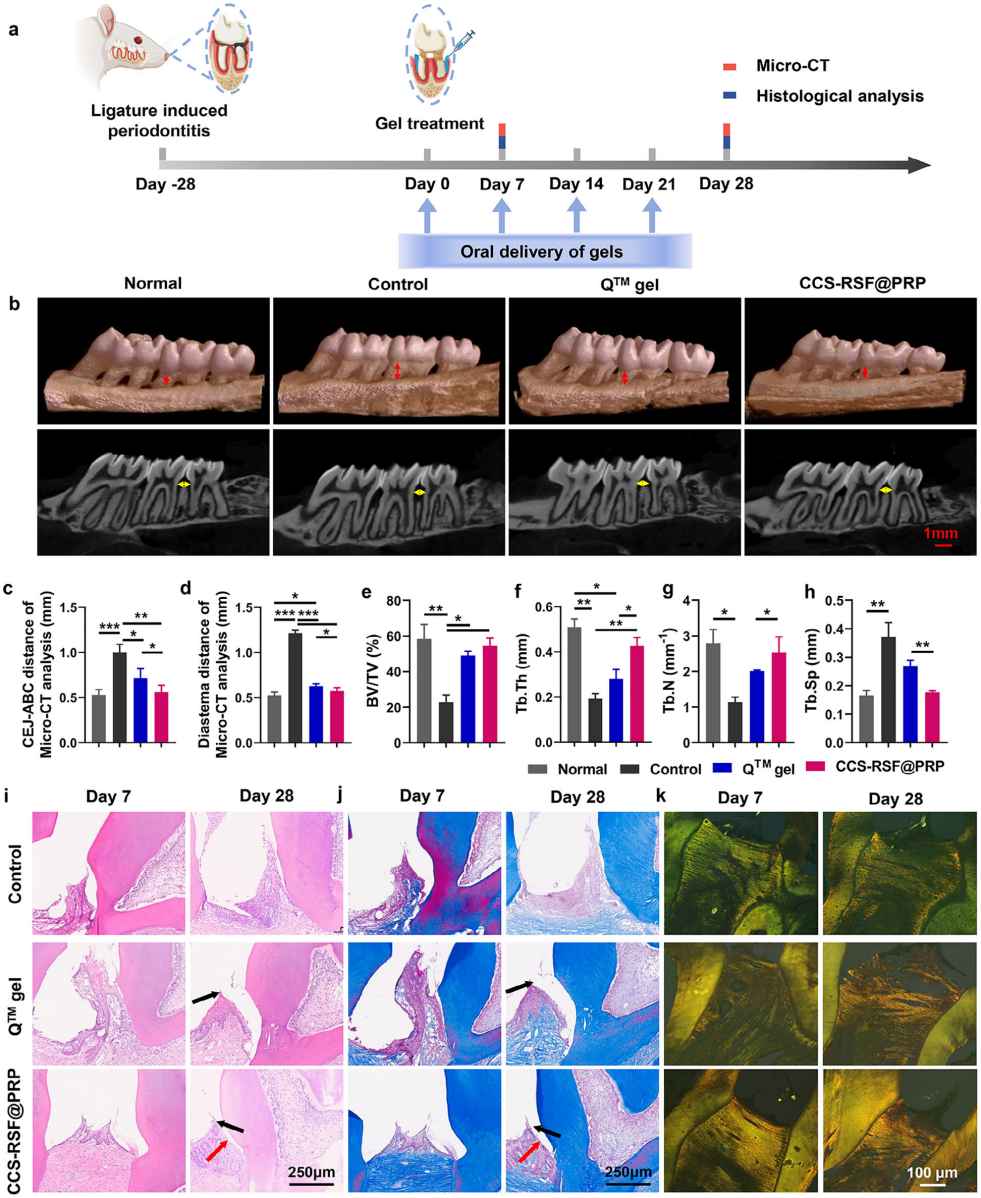
CCS‐RSF@PRP hydrogels promoted the periodontium regeneration for periodontitis treatment. a) Schematic illustration of periodontitis establishment in rats and the protocol for treatment regimen and Micro‐CT and histological analysis. b) Typical 3D micro‐CT images of teeth and periodontal tissues of mice. The red lines with double arrowheads indicate the distance from the cementoenamel junction (CEJ) to the alveolar bone crest (ABC) (termed as CEJ‐ABC). The yellow lines with double arrowheads indicate the diastema distance of maxillary second molar teeth. Quantitative analysis of alveolar bone absorption, including c) CEJ‐ABC, d) diastema distance of maxillary second molar teeth, e) BV/TV, f) Tb.Th, g) Tb.N and h) Tb.Sp (n = 5). i) H&E, j) Masson, and k) Sirius red staining of periodontal tissues on Day 7 and Day 28 after treatment. Black and red arrow points to the interdental papillae, and junctional epithelium, respectively.

The therapeutic effect of CCS‐RSF@PRP hydrogel was tested in a rat periodontitis model around the maxillary second molar teeth (M2). Following periodontal disease induction, CCS‐RSF@PRP hydrogels were directly injected into the periodontal pocket and immunomodulation and tissue regeneration were assessed at cellular and tissue levels (**Figure** **4**a; Figure S27, Supporting Information). Commercial hydrogel Q was used as the positive control. First, the retention time of CCS‐RSF@PRP hydrogels in periodontal pocket was investigated by non‐invasive fluorescence imaging. CCS‐RSF@PRP hydrogels labeled with Cy5 were injected into the lesion of periodontitis and fluorescence images were recorded at scheduled time points (Figure S28a,b, Supporting Information). It was shown that fluorescence intensity was gradually decreased and 92.1% of initial hydrogel was degraded after 7 days. Therefore, the hydrogel was administered every 7 days in periodontitis treatment. H&E staining showed no significant changes in pathological structure of major organs including the heart, liver, spleen, lungs, and kidneys, demonstrating the biosafety of CCS‐RSF@PRP hydrogel for application in vivo (Figure S28c, Supporting Information). In addition, we injected CCS‐RSF@PRP hydrogel under the oral mucosa to test its biosafety (Figure S29, Supporting Information). Compared with CCS‐RSF@PRP (10 v/v%) hydrogel, there was no aberrant infiltration of inflammatory cells in oral mucosa in CCS‐RSF@PRP (100 v/v%) hydrogel group, while the collagen deposition at the injection site was less. All of these results indicated the safety of CCS‐RSF@PRP hydrogel without obvious immunologic rejection in vivo.

Micro‐computed tomography (micro‐CT) was commonly used to evaluate bone resorption in periodontitis. As shown in Figure 4b–d, at day 28 post‐treatment, micro‐CT scanning revealed that compared with the normal rats, the alveolar bone of rats in the control group was obviously absorbed (CEJ‐ABC distance was large), the alveolar crest was reduced, the root forks were exposed and both sides were connected, and the diastema distance between the second molar and the adjacent molar was significantly enlarged. Compared with the commercial Q gel group, after being treated with CCS‐RSF@PRP, alveolar bone formation increased significantly, and root forks were almost completely covered by bone. Especially, after 28 days post‐treatment, in CCS‐RSF@PRP treatment group, the alveolar crest was the highest among the three groups, and molar space was close to normal teeth, suggesting that CCS‐RSF@PRP hydrogel could effectively inhibit the absorption of alveolar bone. Further quantitative assessment by measuring bone volume fraction (BV/TV), trabecular thickness (Tb. Th), trabecular number (Tb. N), and trabecular separation (Tb. Sp) also confirmed the beneficial outcome with the treatment of CCS‐RSF@PRP hydrogels (Figure 4e–h).

To further confirm the therapeutic efficiency in periodontal tissue fusion and repair, H&E, Masson, and Sirius Red staining were performed to evaluate the periodontium regeneration. As shown in Figure 4i,j, in the control group, there was decreased adhesion at the ligation site, absence of interdental papillae structure between molar teeth, shrinkage of junctional epithelium, a large number of infiltrating inflammatory cells and decreased height of alveolar bone, indicating persistent inflammation and serious resorption of alveolar bone in the control group. In the commercial Q gel group, the junctional epithelium was not tightly attached and the epithelium was thicker, the inflammatory cells in the gingival groove were less infiltrated, which meant that the inflammation was partly controlled. However, the alveolar bone in the commercial Q gel group was not improved compared with a control group. In the CCS‐RSF@PRP group, inflammatory cells were less infiltrated, and the epithelium formed was similar to the normal epithelium, with normal interdental papillae and alveolar crest height. Masson staining analysis (Figure 4j) showed that the CCS‐RSF@PRP group showed more orderly and dense collagen deposition (blue reticular fiber structure), which was closely attached to the root of teeth than the commercial Q gel group. In the control group, the collagen fiber bundle was broken and collagen deposition was disordered. Furthermore, we carried out Sirius red staining to further specially distinguish collagen type in periodontal soft tissue (Figure 4k), in which collagen type I (C‐I) was stained as yellow or red, while collagen type III (C‐III) was stained as green. C‐III is synthesized mainly in the initial stage of wound healing and then gradually replaced by C‐I in 2–3 weeks after the start of wound healing. And C‐I found in most connective tissues is the most abundant type of collagen in periodontal tissue and its expression is linked to soft and hard periodontal tissue turnover. After CCS‐RSF@PRP hydrogel treatment for 28 days, the mature collagen type I was significantly more dominant than collagen type III compared to that of other groups (Figure S30, Supporting Information), indicating the accelerated collagen synthesis, which was conducive for the repair and regeneration of periodontal soft tissue. These results indicated that CCS‐RSF@PRP hydrogel can effectively promote tissue fusion and repair, and improve bone regeneration without using additional drugs, cytokines or cells, which is beneficial to the tissue repair of periodontitis. It should be noted that on account of the stimulation of ligature wire during the whole process, the interdental papillae and junctional epithelium were not completely restored in the experimental groups.

### 2.4 Inflammation Microenvironment of Periodontitis Modulated by CCS‐RSF@PRP Hydrogels

**Figure 5:**
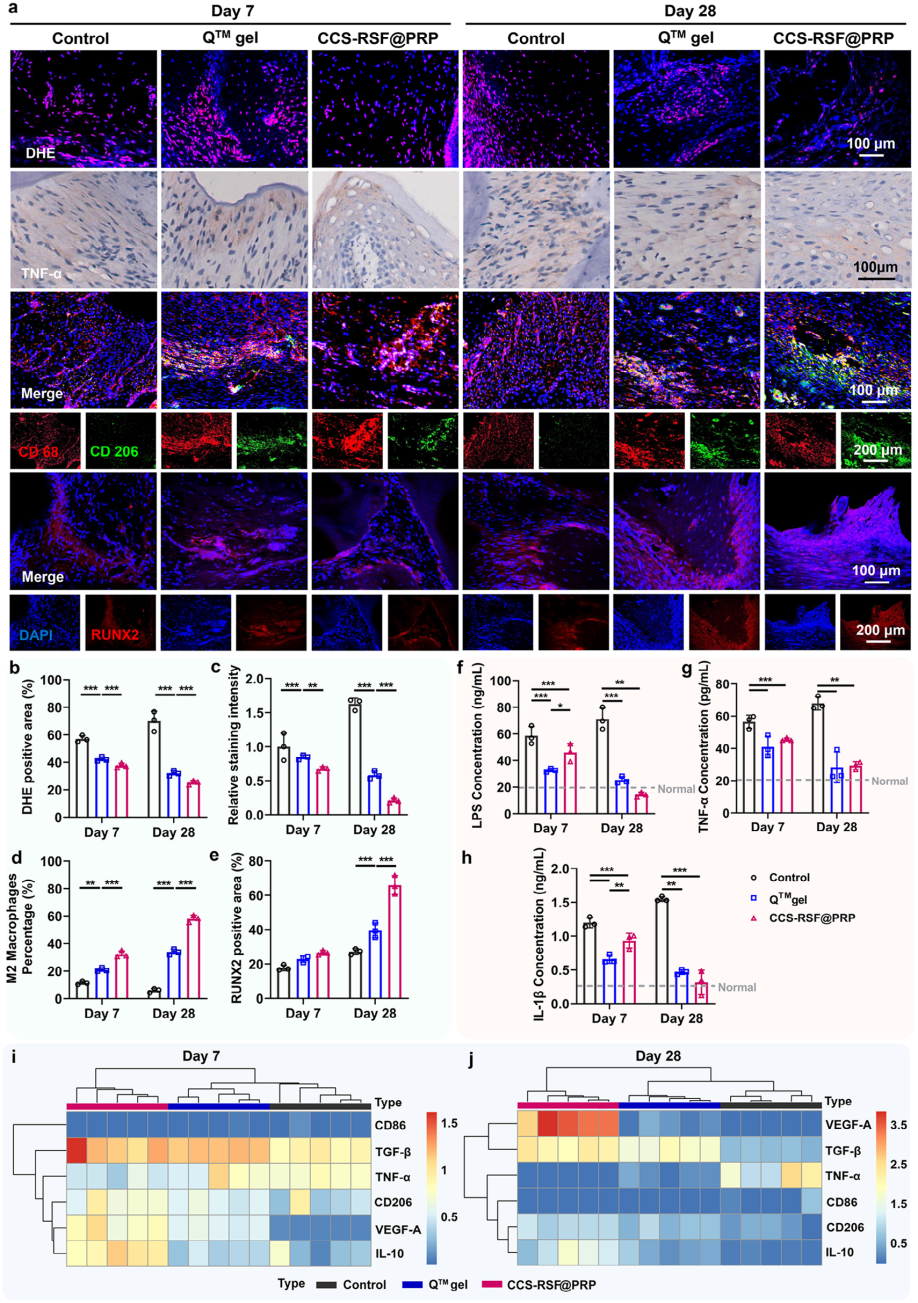
CCS‐RSF@PRP hydrogels modulated the chronic inflammation microenvironment of periodontitis. a) Representative images of DHE staining (red: DHE, blue: DAPI), immunohistochemical staining of TNF‐α, immunofluorescence staining of CD206/CD68 and RUNX2 (red: RUNX2, blue: DAPI) on day 7 and day 28 after treatment. b) Statistical data of DHE positive area (%) (n = 3). c) Quantification of the relative content of TNF‐α in tissue (n = 3). d) Statistical data of the percentage of M2 macrophages (%) (n = 3). e) Statistical data of RUNX2 positive area (%) (n = 3). f‐h) Quantification of inflammatory cytokines LPS, IL‐1β, and TNF‐α in periodontium at the surgical site (n = 3). i‐j) Relative mRNA expression level of anti‐inflammatory cytokines (CD206, VEGF‐A, TGF‐β, and IL‐10) and pro‐inflammatory cytokines (TNF‐α and CD86).

To further certify the healing mechanism and the impact of CCS‐RSF@PRP hydrogels on the chronic inflammatory microenvironment of periodontitis, the level of multiple inflammatory mediators in periodontium was evaluated. As shown in **Figure** **5**a,b, ROS level in CCS‐RSF@PRP hydrogel group, represented by the DHE positive area, was significantly lower than that in the control group and commercial Q group at day 7 and 28, suggesting the ROS scavenging capability of CCS‐RSF@PRP hydrogels in vivo. Figure 5a,c showed that the expression level of pro‐inflammatory TNF‐α was highly elevated in the control group, demonstrating the excessive inflammatory response during the progression of periodontitis. After the treatment of CCS‐RSF@PRP hydrogels, the expression level of TNF‐α was significantly down‐regulated compared to the control and Q group, preliminarily proving that CCS‐RSF@PRP hydrogel can effectively relieve the inflammatory microenvironment. Then, the infiltration and polarization of macrophages in the periodontium after treatment were identified by immunofluorescence staining. CD206 and CD68 were chosen as a surface marker of M2 macrophages and macrophages of all subsets, respectively. Figure 5a showed that there were more M2 macrophages distributed in the periodontal tissues of CCS‐RSF@PRP hydrogels group than other groups after 7‐ and 28‐day treatment. The percentage of M2 macrophages (Figure 5d) in the periodontal tissues of CCS‐RSF@PRP hydrogels group was continually increased from 7 to 28 days post‐treatment, which was significantly higher than that of the control and commercial Q group, indicating that CCS‐RSF@PRP hydrogels potently stimulated the macrophages polarization to M2 type in vivo. In addition, RUNX2, an osteogenic marker, is an important osteogenic transcription factor that can bind to osteoblast‐specific cis‐acting element (OSE) 2 in the promoter region of osteogenic genes. Results in Figure 5a,e showed that CCS‐RSF@PRP group displayed a more widespread distribution of RUNX2‐positive cells (red fluorescence) in contrast to control group and Q gel group. Semiquantitative analysis of RUNX2‐positive area to tissue area in vision field (RUNX2^+^/Tissue) showed that the positive ratio in CCS‐RSF@PRP group was ≈65.89%, which was significantly higher than that in other groups (Figure 5e). These results indicated that CCS‐RSF@PRP hydrogel inhibited bone resorption and stimulated the bone formation, mainly owing to the immunoregulatory function of CCS‐RSF@PRP in remitting deteriorative inflammation environment and sustained release of growth factors, thus beneficial to inhibit alveolar bone loss and beneficial outcome of periodontitis treatment.

Furtherly, periodontium at the surgical site was extracted to quantify the level of endotoxin LPS and inflammatory cytokines including TNF‐α and IL‐1β at day 7 and 28 post‐treatment. As shown in Figure 5f–h, compared with the control group, the levels of LPS, TNF‐α, and IL‐1β in the periodontium at day 28 were significantly downregulated after treated with CCS‐RSF@PRP hydrogels, indicating the hydrogel effectively neutralized these proinflammatory mediators, thereby regulating the repeated and chronic inflammation and improving the tissue regeneration of periodontitis. Moreover, macrophage polarization‐associated RNA expression in the periodontium was further detected through RT‐qPCR. As shown in Figure 5i,j, treatment with CCS‐RSF@PRP hydrogels not only decreased the mRNA expression of CD86 and proinflammatory cytokine of TNF‐α, but also increased the mRNA expression of CD206 and anti‐inflammatory cytokines such as VEGF‐A, TGF‐β and IL‐10, indicating that CCS‐RSF@PRP hydrogels could elicit a pro‐reparative immune response through harnessing the M2 phonotype macrophage polarization.

**Figure 6:**
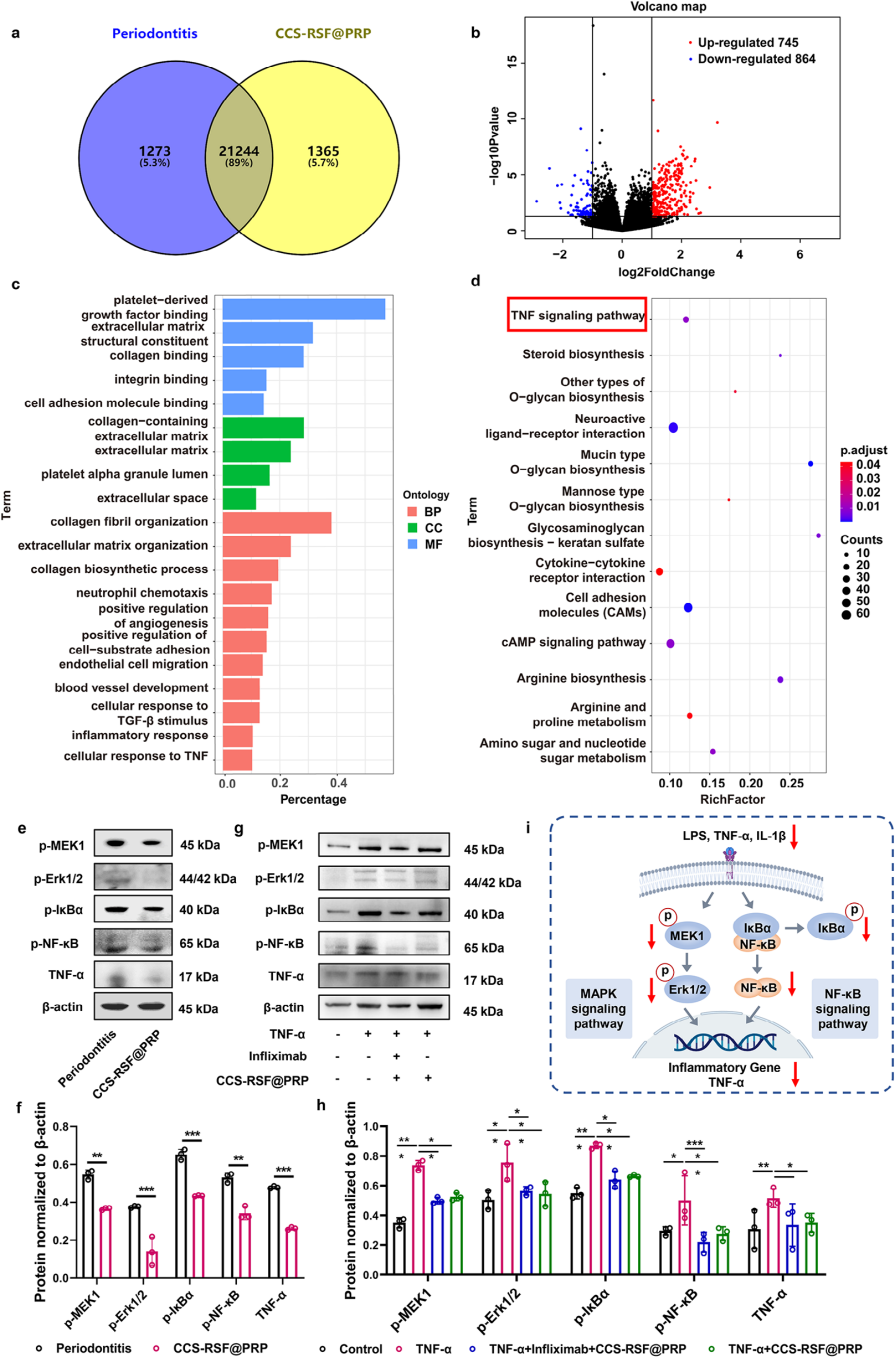
Mechanism analysis of periodontitis therapy with CCS‐RSF@PRP hydrogels. a) Venn diagram of the number of differentially expressed genes (DEGs). b) Volcano plots showing upregulated and downregulated DEGs. c) Gene ontology analysis of top 20 enriched terms between periodontitis group and CCS‐RSF@PRP hydrogels group. BP, biological processes; CC, cellular components; MF, molecular functions. d) KEGG pathway analysis demonstrating the top 13 signal pathways enriched by DEGs. e, f) Protein expression level of p‐MEK1, p‐Erk1/2, p‐IκBα, p‐NF‐κB and TNF‐α in periodontium of periodontitis and CCS‐RSF@PRP treatment group determined by western blot (n = 3). g, h) Protein expression level of p‐MEK1, p‐Erk1/2, p‐IκBα, p‐NF‐κB and TNF‐α of BMDMs in the control, TNF‐α, TNF‐α+infliximab+CCS‐RSF@PRP and TNF‐α+CCS‐RSF@PRP groups determined by western blot (n = 3). i) The mechanism of CCS‐RSF@PRP hydrogel remitting inflammation of periodontitis through TNF signaling pathway.

To investigate the underlying mechanisms responsible for the therapeutic effects of CCS‐RSF@PRP hydrogels, we performed RNA sequencing analysis of the periodontium. The results revealed a large number of different genes by comparing different samples and 21 244 genes were shared (**Figure** **6**a), including 745 and 864 genes with upregulated and downregulated expression, respectively (Figure 6b). Based on these differently expressed genes (DEGs), we performed gene ontology (GO) enrichment analysis on three gene ontology categories: biological processes (BP), cellular components (CC), and molecular functions (MF) (Figure 6c). The 20 most enriched terms between periodontitis group and CCS‐RSF@PRP hydrogels group included platelet‐derived growth factor binding and extracellular matrix structure constituent in molecular functions, collagen‐containing extracellular matrix and neutrophil chemotaxis in cellular components, positive regulation of angiogenesis, endothelial cell migration and inflammatory response in biological processes, which correlated with the extracellular matrix (ECM) remodeling, polarization of macrophages and immune response. To further evaluate the underlying signal transduction pathways, we performed Kyoto Encyclopedia of Genes and Genomes (KEGG) pathway enrichment (Figure 6d). CCS‐RSF@PRP hydrogels were involved in the regulation of multiple signaling pathways, including TNF signaling pathway, cytokine‐cytokine receptor interaction, and cell adhesion molecules, which is associated with the inflammatory reaction and tissue regeneration, contributing to curing periodontitis disease. Considering that TNF is a key mediator in the inflammatory response and triggers the activation of many pathways, including the NF‐κB and MAPK pathways, we choose TNF signaling pathway for further verification on the inflammatory regulation effect of CCS‐RSF@PRP hydrogel through western blot (Figure 6g). The nuclear transcription factor NF‐κB, known as the key activator of inflammation, and phosphorylated IκB family proteins (IκBα proteins), a key regulator of the classical NF‐κB signaling pathway, were evaluated as its upstream protein in NF‐κB signaling pathway. Extracellular‐signal regulated protein kinase (Erk) subfamilies, one of the most critical subfamilies in MAPK cascade response pathway, were phosphorylated and translocated to the nucleus to regulate a variety of transcription factors after stimulation of the organism, which further promoted the transcription and translation of inflammatory factors and intensified the inflammatory response, and phosphorylated MEK1 protein was evaluated as its upstream protein in MAPK signaling pathway. As shown in Figure 6e,f, western blot analysis showed that the phosphorylation of MEK1, Erk1/2, IκBα and NF‐κB and the expression of TNF‐α were significantly downregulated by CCS‐RSF@PRP hydrogel treatment, compared with that of periodontitis group, indicating that inflammatory regulation effect of CCS‐RSF@PRP hydrogel was potentially transmitted through TNF signaling pathway.

Furthermore, the TNF‐α pathway was verified at a cellular level on BMDMs. TNF‐α was employed as an activator, and infliximab was used as a pathway inhibitor, which is a chimeric monoclonal IgG1 antibody that specifically binds to TNF‐α and blocks the interaction of TNF‐α with TNF‐α receptor 1 (TNFR1) and TNFR2. The expression of related proteins including the phosphorylation of MEK1, Erk1/2, IκBα, NF‐κB, and TNF‐α were investigated by western blot. As shown in Figure 6g,h, compared with control group, the expression of phosphorylated MEK1, Erk1/2, IκBα, NF‐κB, and TNF‐α were significantly downregulated by CCS‐RSF@PRP hydrogel or infliximab treatment, compared with that of TNF‐α treatment. CCS‐RSF@PRP showed comparable ability in inhibiting TNF pathway in comparison with infliximab at a concentration of 40 ng/mL. These data further confirmed that TNF signaling pathway was involved in the inflammatory regulation of CCS‐RSF@PRP hydrogel. Therefore, CCS‐RSF@PRP hydrogels could notably re‐shape the local immune response by neutralizing the multiple proinflammatory mediators of pathogen LPS, ROS, and polarizing macrophages toward M2 type, thus dampening the positive feedback loop of destructive inflammation in periodontitis and promoting the ECM remodeling and tissue regeneration.

## 3 Discussion

As the sixth largest public health problem in the world, severe periodontitis affects 10–15% of the world's population. Current clinical treatments for periodontitis focus on plaque removal and local inflammation control, such as scaling, root planning, and surgical treatments, which attempt to minimize symptoms and prevent disease progression but cannot deracinate chronic inflammation and restore the structures and functions of periodontal tissues for periodontitis patients.^[^ ^6c^ ^]^ Simple mechanical debridement cannot remove the plaque microorganisms in deep periodontal pockets, tortuous pockets, and root forks, thus adjuvant medication is required. The drugs used for the treatment of periodontitis in clinics are mainly divided into antibiotics (tetracycline), non‐steroidal anti‐inflammatory drugs, and biological agents that regulate the host defense response.^[^ ^7e^ ^]^ The clinical use of antibiotics mainly adopts local administration (PERIO, H_2_O_2,_ and chlorhexidine) and systemic administration (metronidazole, etc.). H_2_O_2_ and chlorhexidine are mainly used as local irrigation drugs but are easily affected by saliva and gingival crevicular fluid in the mouth, reducing local drug concentration and shortening drug retention time. Additionally, systemic administration will reduce the concentration of drugs in the periodontal pocket, and also easily induce drug resistance and flora imbalance of strains.^[^ ^27^ ^]^ The hydrogel formulation PERIO can prolong the half‐time of the encapsulated drug, but it only contributes to sterilization and cannot alleviate inflammation or prevent alveolar bone resorption.^[^ ^28^ ^]^ In the past decades, many anti‐inflammatory drugs or cytokines, such as selective cytokine blockers for interleukin‐1 (IL‐1), interleukin‐6 (IL‐6), and tumor necrosis factor‐α (TNF‐α), have been verified in the clinical treatment of a variety of acute and chronic diseases. For example, the use of omega‐3 polyunsaturated fatty acids and low‐dose aspirin reduced the concentration of IL‐1β and IL‐10 in the gingival crevicular fluid, which was beneficial to periodontal regeneration.^[^ ^29^ ^]^ However, these small molecule drugs suffer from rapid diffusion and metabolism in vivo, resulting in a limited therapeutic effect. And overdosing on drugs often result in the risk of adverse effects. The commercial Q gel, which is composed of chitosan hydrogel and menthol with antibacterial and anti‐inflammatory properties, was selected in this work as a positive control for the therapeutic effect evaluation in vivo. But short retention time and the side effects of high concentration of menthol was also inevitable.^[^ ^30^ ^]^ Moreover, periodontitis is a complex chronic inflammatory system involving multiple inflammatory mediators, of which compensatory mechanisms exist between them. A single drug or cytokine only targets a specific inflammatory pathway, which may be limited in periodontitis treatment.

Hemostasis is a physiological mechanism that seals damaged blood vessels to stop bleeding and activates host defense through a series of complex and rapid coagulation reaction mediated by thrombin and activation of platelets, and finally generation of hemostatic blood clots.^[^ ^13^, ^31^ ^]^ Blood clots are mainly composed of activated platelets and fibrin, which represent a viable, dynamic matrix of proteins and cells that not only contribute to hemostasis but also serve as a provisional lattice for incoming inflammatory cells, fibroblasts, and growth factors to further promote tissue repair.^[^ ^32^ ^]^ However, in periodontitis, these clots are highly unstable, which will dissolve rapidly within 24–48 h and are insufficient to counteract chronic inflammation, rendering them unsuitable as standalone therapies for periodontitis.^[^ ^32^ ^]^ Alternatively, PRP, an autologous plasma product with rich growth factors, is widely used in various medical fields due to its simple preparation, autologous origin, and lack of immune rejection risks. But the poor stability and mechanical fragile, rapid degradation rate, and the safety concerns raised by the activation of thrombin are also limited its further application. To structurally and functionally mimic the blood clot, the CCS‐RSF@PRP hydrogel in this work was engineered through structural design and optimization while overcoming these challenges. It has been shown that the positive charge on chitosan and RSF nanofibers may activate PRP without adding exogenous activators.^[^ ^32^, ^33^ ^]^ Chitosan possesses amino groups (‐NH_2_) that protonate (‐NH3^+^) at physiological pH, conferring a positively charged surface. The positively charged surface promotes platelet adhesion, aggregation, and degranulation via electrostatic interactions with negatively charged phospholipids on platelet membranes, thereby releasing growth factors (e.g., PDGF, TGF‐β, VEGF).^[^ ^34^ ^]^ The electrospun silk fibroin nanofibers exhibit a high specific surface area and biomimetic extracellular matrix (ECM) structure. The nanofiber network provides anchoring sites for platelets, facilitating their aggregation and activation of endogenous coagulation pathways. The β‐sheet conformation of silk fibroin mimics the mechanical properties of collagen fibers, activating integrin receptors on platelet surfaces.^[^ ^32^, ^35^ ^]^ Therefore, the elaborate integration of chitosan, RSF, and PRP should be a facile strategy to recapitulate the physiochemical cues and biofunctions of natural blood clot. The prepared CCS‐RSF@PRP hydrogel has a hierarchical fibers‐interwoven network structure distributed with activated platelets (Figure 1e,f), which was structurally similar to the native blood clot. And it also holds merits including injectability (Figure S5c, Supporting Information), self‐healing capability (Figure 1d), adjustable mechanical properties (Figure 1c), well stability, and extended shelf life (Figure S11a–c), providing an ECM‐like pro‐regenerative microenvironment after injection in periodontium. Functionally, in addition to the known function of enriching and releasing growth factors, our hydrogel also could neutralize inflammatory mediators to inhibit excessive inflammation and promote the regeneration of periodontal tissue for periodontitis treatment. Therefore, through integrating PRP, we fabricated a functionalized artificial blood clot material CCS‐RSF@PRP hydrogel to mimic and expand the structure and function of native blood clot for periodontitis treatments.

To verify the effect in inflammatory regulation of CCS‐RSF@PRP hydrogel, we choose macrophages as the representative of immune cells in our study. Macrophages, as an important part of innate immunity, are found in the gingival epithelium, lamina propria and perivascular tissues in periodontal tissues. The number of macrophages in the gingival tissues of periodontitis patients is significantly higher than that in healthy gingival tissues^[^ ^36^, ^37^, ^38^ ^]^ Macrophages are the main source of inflammatory factors involved in periodontal tissue destruction. A variety of periodontal pathogens can activate periodontal macrophages to pro‐inflammatory state, such as Porphyromonas gingivalis (*P. g*), as transmitted through TLR2 and TLR4 pathway, so that they can produce a large amount of pro‐inflammatory cytokines including TNF‐α, IL‐1β and IL‐10, et al. Therefore, macrophages are critical to the inflammatory response and the onset of periodontitis. Additionally, macrophages can be manifested as different subtypes in different microenvironments and play unique functions, affecting the genesis and development of periodontitis. According to the activation pathway and function, macrophages can be roughly divided into two categories: classical activation type (M1, pro‐inflammatory macrophages) and alternative activation type (M2, anti‐inflammatory macrophages). M1 macrophages mainly participate in the progression of periodontitis and the destruction of periodontal tissue. Oppositely, M2 macrophages primarily participate in the regeneration and repair stage of the damaged tissue to eliminate inflammatory stimulation. Hence, macrophages are involved in all stages of periodontitis, from inflammation response to repair and regeneration.^[^ ^36^, ^38^ ^]^ The correction of imbalanced macrophage homeostasis caused by the progression of chronic inflammation may provide a new pattern for the treatment of periodontitis. Herein, we selected BMDMs as a representative of immune cells to study the neutralization of LPS (Figure 2m,n; Figures S20–S23, Supporting Information), the effects on M1 and M2 polarization of macrophages (Figure 3e–n; Figures S25 and S26, Supporting Information), and the immunomodulatory effects on periodontal inflammatory environment (Figure 5a,d). It was found CCS‐RSF@PRP hydrogel could effectively neutralize LPS, inhibit the M1 polarization of macrophages, and promote M2 polarization, which is devoted to blocking the chronic inflammatory feedback loop in periodontitis and facilitating periodontium regeneration.

Concerning the anti‐inflammation mechanism, we have clarified that CCS‐RSF@PRP could modulate inflammation from the following aspects, including pro‐inflammatory cytokine neutralization, ROS scavenge, macrophage M2 polarization, as well as TNF‐α pathway inhibition. Tissue regeneration is a complicated and multiplex process involving a dynamic inflammatory microenvironment. Chronic inflammation is characterized by the prolonged response to inflammatory signals, accompanied by continuous recruitment of lymphocytes, monocytes, and macrophages, which were activated by multiple inflammatory mediators including ROS, bacterial components, genes, and cytokines/chemokines. This work suggested the artificial blood clot hydrogel could specifically bind and neutralize multiple proinflammatory mediators to alleviate the inflammation response by blocking their downstream signaling activation. Our results demonstrated that the CCS‐RSF@PRP hydrogel could downregulate the ROS level through dopamine chemistry (Figure 2a–h), detoxify LPS by thrombin (Figure 2i–n), and neutralize proinflammatory factors including TNF‐α, IL‐1β and IFN‐γ by the receptors on the platelets membrane (Figure 3b–d and Figure S24, Supporting Information). This binding and neutralizing effect of the hydrogel could detoxify these inflammatory mediators by consuming them or dampening the binding of cytokines with receptors and downstream signaling pathways.^[^ ^12^, ^39^ ^]^ In particular, TNF signaling pathway was enriched with CCS‐RSF@PRP hydrogel treatment (Figure 6d–h), certificating the inhibitory effect of the hydrogel on the activation of inflammation‐associated signaling pathways. However, due to the complex relationships between inflammatory mediators and cell interactions, beyond the obtained signaling pathway and activated receptors such as CD40, CD62P, and CD121b, there should be other inflammatory pathways involved in the immunomodulation effect of the hydrogel. For instance, the downregulation of intracellular ROS has been proven to be effective in suppressing the activation of inflammatory cells, and the release of anti‐inflammatory cytokines could also contribute to protect cells and tissues from oxidative damage.^[^ ^22^, ^40^ ^]^ Since CCS‐RSF@PRP hydrogel comprises anti‐inflammatory growth factors, these factors should have potentially performed a function of reliving inflammation during tissue regeneration, via regulation of the proliferation, differentiation, and activation of immune cells, or association with immune response.^[^ ^41^ ^]^ Moreover, other inflammatory pathways may interfere with TNF signaling pathway in the reduction of an inflammatory response by CCS‐RSF@PRP hydrogel, including TNF‐α downstream JNK signaling pathway, IL‐1β downstream JAK/STAT signaling pathway, and IFN‐γ downstream PI3K‐Akt pathway.^[^ ^42^ ^]^ And this work primarily focuses on the inflammatory response of macrophages. However, beyond macrophages, other populations of immune cells, including neutrophil, dendritic cells, and T cells also play crucial roles in the progression of periodontitis. At the initial stage of periodontitis, antigen‐presenting cells, such as macrophages and dendritic cells at the inflammatory site would activate naive T cells, and pro‐inflammatory T cells (Th1 and Th17) further secrete pro‐inflammatory cytokines (IFN‐γ, TNF‐α, IL‐17) to deteriorate the inflammatory response.^[^ ^43^ ^]^ The polarization and proliferation of these cells may also be involved in the treatment of periodontitis. Therefore, the interactions among biomaterials, pro‐inflammatory cytokines, and other immune cells, as well as intervention against various possible inflammatory signaling pathways, need to be fully understood in future studies to confirm the significant role of artificial blood clot hydrogel in the immunomodulation effect. Such an anti‐inflammatory hydrogel may also be applied in treating other chronic inflammatory disease models, such as arthritis and diabetic ulcer.

In the preparation of CCS‐RSF@PRP hydrogel, the extraction of rat PRP is allogeneic, which was based on the following considerations. First, the process of autologous blood extraction will cause physical and mental damage to rats, resulting in individual differences in animal models, which makes it difficult to ensure that CCS‐RSF@PRP hydrogel has an accordant therapeutic effect on periodontitis. Second, individual blood heterogeneity may cause differences in CCS‐RSF@PRP hydrogel, thus affecting the experimental results. In comparison, homologous PRP has the advantages of convenient uniformity, standardized preparation, low cost, and ready supply without considering the influence of autologous blood collection. Moreover, the current researches have not found any adverse reactions of allogeneic blood, which can meet the needs of more patients.^[^ ^44^ ^]^ And numerous clinical and pre‐clinical studies have used allogeneic blood as a therapeutic agent.^[^ ^45^ ^]^ There are still some limitations in this work. Although the PRP in this study was prepared by rat‐isolated PRP, which may limit its further application to treat human disease. The source of PRP is an important issue that needs to be considered in future preclinical research. The treatment effects and immune rejection from different sources of PRP also need to be further considered. For further translational studies, allogeneic PRP can be obtained from the peripheral blood of one or more healthy donors, and preserved until application. Additionally, the same batch of PRP may be used to treat a single patient over time or to treat several different patients. Moreover, the potential effects of salivary enzymes on PRP activity should be taken into account when it is applied for periodontal treatment. It has been reported that a few inflammatory proteases in salivary enzymes, such as matrix metalloproteinase, gingipains, and trypsin, may degrade released factors from PRP, thus impairing PRP bioactivity.^[^ ^46^ ^]^

Overall, we successfully engineered an off‐the‐shelf artificial blood clot hydrogel with potential clinical translation for treating periodontal disease. CCS‐RSF@PRP hydrogel was prepared by integrating PPR, CCS, and RSF nanofibers, of which the PRP could be activated in situ without adding exogenous activators evidenced by numerous pseudopods of platelets and fibrous structure in the hydrogel network. More importantly, in order to intervene in the repetitive inflammation of periodontitis, the catechol group was introduced in CCS‐RSF@PRP hydrogels with inherent ROS scavenging capability to relieve oxidative stress injury to the host periodontal tissue. And the diversity of platelet membrane receptors in activated PRP was confirmed to selectively capture and bind LPS and multiple chemo‐ and cytokines including TNF‐α, IFN‐γ and IL‐1β, and drive the macrophages polarization toward M2 phenotype, which could synergistically block the chronic inflammatory feedback loop and regulate immune homeostasis of periodontitis. In a rat periodontitis model, CCS‐RSF@PRP hydrogels effectively inhibited bone loss and promoted periodontal tissue fusion and repair, attributing that CCS‐RSF@PRP hydrogel could rebuild a pro‐reparative immune microenvironment through neutralizing the multiple proinflammatory mediators of pathogen LPS, ROS, pro‐inflammatory factors and polarizing macrophages toward M2 type. Our results also highlight the importance of delicately designed biomaterials to neutralize multiple proinflammatory mediators in dampening the destructive inflammation of periodontitis. Findings presented in this work suggest the off‐the‐shelf and biomimetic CCS‐RSF@PRP hydrogels are promising for regenerative periodontal therapy, as well as for other inflammatory diseases such as arthritis, nerve injury, and myocardial infarction.

## 4 Experimental Section

### Materials

Chitosan, 1‐(3‐Dimethylaminopropyl)‐3‐ethylcarbodiimide hydrochloride (EDC), N‐Hydroxysuccinimide (NHS), Hydrocaffeate (HCA) were purchased from Aladdin Chemical Reagents Co., Ltd. (Shanghai, China). Na_2_CO_3_, LiBr, and CaCl_2_ were purchased from HEOWNS (Tianjin, China). Thrombin was purchased from Solarbio (Beijing, China). IL‐4 and recombinant mouse monocyte colony‐stimulating factor (M‐CSF) were purchased from Pepro Tech (Rocky Hill, NJ, USA). LPS was purchased from Beyotime Biotechnology (Shanghai, China). Fluorochrome‐labeled monoclonal antibodies (CD86, CD206, MHC II, F4/80) were received from eBioscience (San Diego, CA, USA). Primary antibodies to Arg‐1, iNOS, and β‐actin, and secondary antibodies were purchased from Abcam (Cambridge, UK).

### Fabrication and Characterization of Hydrogel Constructs

The detailed preparation process of CCS, RSF, and PRP was illustrated in Supporting Information. CCS and RSF composite glycoprotein hydrogel (termed as CCS‐RSF hydrogel) was formed through the intermolecular interaction by simply dissolving CCS in DDW (2 wt%) and dispersing the desired amount of RSF in the above solution. The gelation occurred in 1–2 min upon vortex. Hydrogel loaded with PRP (termed as CCS‐RSF@PRP hydrogel) was fabricated by briefly mixing PRP solution with CCS‐RSF hydrogel and then co‐incubated at 37 °C for 30 min. The composition of hydrogel was evaluated by FTIR spectroscopy (BIO‐RAD FTS3000).

### Morphology and Rheology Properties of Hydrogels

The microstructures of hydrogels and nanofibers were examined by the scanning electron microscope (SEM, S‐4800, Hitachi, Japan) and Confocal Laser Scanning Microscope (CLSM) (TCS SP5II, Leica, Ernst‐Leitz‐Strasse, Germany). Rheological measurements were performed on CCS‐RSF@PRP hydrogels with varied amounts of RSF nanofibers and PRP using an AR 2000ex rheometer (TA). Further details are provided in the Supporting Information.

### Cytocompatibility Evaluation of Hydrogels

The cytotoxicity of hydrogels was evaluated using cell counting kit‐8 assay kit (CCK‐8, Solarbio, CA1201) and live/dead assay kit (Solarbio, CA1630). Further details are provided in the Supporting Information. To visualize cell proliferation in hydrogels, GECs cells at a density of 1 × 10^5^ cells per well were encapsulated in hydrogels in confocal dishes. After incubating for 1 day and 3 days, the culture medium was carefully removed and then evaluated by live/dead assay kit and observed by CLSM.

### ROS Scavenging Capability of CCS‐RSF@PRP Hydrogel

H_2_O_2_, 1,1‐diphenyl‐2‐picrylhydrazyl (DPPH) free radical, superoxide anion, and hydroxyl free radical scavenging assays were conducted to investigate the ROS scavenging properties of CCS‐RSF@PRP hydrogel. A reactive oxygen species assay kit (2,7‐dichlorodihydrofluorescein diacetate, DCFH‐DA; S0033S, Beyotime, China) was used to test the cellular ROS scavenging activity. The ROS levels were measured using CLSM and flow cytometry. Furthermore, the effects of H_2_O_2_ on the cell viability and apoptotic of GECs were evaluated through CCK‐8 assay and flow cytometry, respectively. Further details are provided in the Supporting Information.

### LPS Neutralization In Vitro

To evaluate the effect of LPS on cell morphology, GECs (1 × 10^4^ per well) were seeded on confocal microscopic dish and cultured for 24 h. Then, GECs were treated with LPS‐*E. coli* (100 µg/mL) (Solarbio, L8880) and LPS + CCS‐RSF@PRP for 24 h. The group without treatment was used as a control group. Finally, the treated cells were washed three times with cold PBS, fixed with paraformaldehyde, and then stained with Alexa Fluor 555‐conjugated phalloidine (Beyotime, C2203S) and DAPI (Beyotime, C1005) to label the F‐actin and cell nuclear, respectively. Images were captured using a CLSM. The cell viability was quantified using CCK‐8 assay kit and TNF‐α cytokine concentration in the supernatant was tested by ELISA (CUSABIO, CSB‐E09315h) as above.

Macrophage polarization assay was also conducted to evaluate LPS neutralization with CCS‐RSF@PRP. Bone marrow‐derived macrophages (BMDMs) were isolated from C57BL/6 mice (6 weeks old, Vital River Laboratory, China) according to the previous report.^[^ ^47^ ^]^ BMDMs were seeded in a 6‐well plate at the density of 4 × 10^5^ cells per well. Then, LPS‐*E. coli* (Solarbio, L8880) and LPS‐*P. g* (InvivoGen) (40 ng mL^−1^) with or without CCS‐RSF@PRP in fresh culture medium was added and incubated for 48 h. After incubation, cells were collected by centrifugation and washed with PBS. Treated cells were stained with PE‐labeled F4/80 antibody, FITC‐labeled anti‐CD86 antibody, and APC‐labeled CD206 antibody, and then analyzed by flow cytometry (C6, BD, USA).

### Macrophage Activation by Hydrogels

BMDMs were seeded in confocal dishes at the density of 1 × 10^5^ cells per well and treated by diverse medium. Cells cultured in medium supplementing with 40 ng mL^−1^ IL‐4 were used as positive control. After incubation for 48 h, BMDMs were washed twice with cold PBS and stained with FITC‐conjugated phalloidine (CA1620, Solarbio) and DAPI to label the F‐actin and cell nuclear, respectively. Characteristic surface biomarker APC‐labeled CD206 was used to identify M2‐type macrophages. The stained BMDMs were then visualized using a CLSM and three randomly captured images were used to measure elongation using the ImageJ software.

Flow cytometry was also applied to identify the types of macrophages that had been treated with different medium. Treated cells were stained with PE‐labeled F4/80 antibody, FITC‐labeled anti‐MHC II antibody, and APC‐labeled CD206 antibody according to the manufacturer's guidelines, and then analyzed by flow cytometry to further identify the macrophages polarization.

The expression level of mRNA and protein related to M1 and M2 macrophages were tested by real‐time quantitative polymerase chain reaction (RT‐qPCR) and western blot (WB), respectively. Further details are provided in the Supporting Information.

### In Vivo Experiment

All animal procedures were performed in accordance with the Guidelines for the Care and Use of Laboratory Animals of Peking Union Medical College and experiments were approved by the Animal Experiments and Ethics Review Committee of the Institute of Radiation Medicine, Chinese Academy of Medical Sciences (IRM‐DWLL‐2023065). Forty age‐matched male Sprague Dawley (SD) rats were randomly divided into four groups. The specific groups were as follows: the normal group (healthy rats), the control group (periodontitis without treatment), the Q gel group (periodontitis treated with the commercial Q gel, which is mainly composed of chitosan and menthol, endowing it with antibacterial and anti‐inflammatory effect) and the CCS‐RSF@PRP group (periodontitis treated with the CCS‐RSF@PRP hydrogel). A ligature procedure was performed to induce periodontitis in rats, in which a retentive ligature was implemented around the maxillary second molar teeth (M2). After periodontitis in rats was induced by ligation for four weeks, 100 µL of CCS‐RSF@PRP hydrogel and Q gel was locally injected into the periodontal pockets every 7 days. Animals were euthanized on day 7 and day 28 for further analysis. Micro‐CT was conducted 3D reconstruction to estimate tissue destruction. Inflammatory factors (LPS, TNF‐α, and IL‐1β) concentration and M1, M2 macrophages‐related gene expression, including CD206, VEGF‐A, TGF‐β, IL‐10, TNF‐α and CD86 in experimental periodontium were measured through ELISA kit and RT‐qPCR, respectively. Further details are provided in the Supporting Information.

### Transcriptome Sequencing and Data Analysis

The periodontium in periodontitis and CCS‐RSF@PRP hydrogel groups were treated with TRIzol reagent (Invitrogen, USA) and stored at −80 °C before sequencing. RNA sequencing was performed using the TIANSeq Fast RNA Library Kit (Illumina). Gene ontology and KEGG pathway enrichment analyses were performed using the TIANGEN Platform.

### Statistical Analysis

Data were presented as mean ± standard deviations (SDs). Student's t‐test or one‐way ANOVA was used to assess the difference between two groups or multiple groups, respectively. Statistical significance is denoted by ^*^ *p* < 0.05, ^**^ *p* < 0.01, and ^***^ *p* < 0.001.

## Conflict of Interest

The authors declare no conflict of interest.

## Supporting information



Supporting Information

## Data Availability

The data that support the findings of this study are available from the corresponding author upon reasonable request.
