# Supplementary material for: An Off‐the‐Shelf Artificial Blood Clot Hydrogel Neutralizing Multiple Proinflammatory Mediators for Pro‐Regenerative Periodontitis Treatment
Source: Adv Sci (Weinh). 2025 May 28;12(31):e04106. doi: 10.1002/advs.202504106 (PMC12376679; doi:10.1002/advs.202504106)
Supplement: Supplementary file 1 — Supporting Information [file ADVS-12-e04106-s001.docx]

**An Off-the-shelf Artificial Blood Clot Hydrogel** **Neutralizing Multiple Proinflammatory Mediators for** **Pro-regenerative Periodontitis Treatment**

*Yini Huangfu, Zhezhe Zhao, Xiang Liu, Jingrong Wang, Yufeng Zhang, Tingting Lan, Yonglan Wang, Chenxuan Wu*, Ju Zhang, Pingsheng Huang*, Chuangnian Zhang, Anjie Dong, Zujian Feng*, Deling Kong, Weiwei Wang**

Weiwei Wang, Email: wwwangtj@163.com, wangww@bme.pumc.edu.cn

Zujian Feng, Email: fengzj@bme.pumc.edu.cn

Pingsheng Huang, Email: sheng1989.2008@163.com

Chenxuan Wu, Email: cwu04@tmu.edu.cn

**This PDF file includes:**

Supporting text

Figures S1 to S29

Tables S1

**Synthesis of Catechol-Chitosan (CCS).** CCS was synthesized using standard EDC-NHS chemistry. Briefly, 60 mL of 1 wt% chitosan solution was prepared with HCl solution (pH 2.5). HCA (0.3 g) and EDC (0.7 g) with NHS (0.5 g) were dissolved separately in 15 mL ethanol and double distilled water (DDW). The two solutions were mixed and slowly added to chitosan solution under stirring. The pH of the solution was then adjusted to 4.5-5.0 to prevent the oxidation of catechol groups and reacted at room temperature for 18 h under constant stirring. Afterward, the reactant solution was purified using a dialysis membrane (cutoff 8000 Da) against acidified DDW (pH 5.0, HCl) for 2 days and then lyophilized. The catechol conjugation was then characterized by 1HNMR spectrometer (AVANCE III 400 MHz, Bruker, Swiss) and UV-vis spectra (Varian, Santa Clara, CA), respectively. The substitution degree of catechol groups (defined as the moles of catechol per mole of glucosamine units) was calculated according to the absorbance-concentration standard curve of HCA solution ($\text{y=0.2651}\ln\left( \text{x} \right)\text{-0.6935, }\text{R}^{\text{2}}\text{=0.9949}$) fitting on the basis of UV-vis spectra.

**Preparation of Regenerated Silk Fibroin (RSF) Nanofibers via Electrospinning.** Firstly, 4.0 g of bombyx cocoons were boiled in 500 mL of 0.5 wt% Na_2_CO_3_ solution for 30 min to remove the sericin protein and then rinsed with distilled water. The degummed silk fibroins were dried in a ventilated place for 12 h. Subsequently, the dried silk fibroins were dissolved in 100.0 mL of 9.0 mol/L LiBr solution at 40 ^o^C for 2 h and then diluted with deionized water. Centrifuge to remain the precipitate. Finally, the precipitate was dialyzed for 48 h and then lyophilized to get the RSF sponges.

RSF solutions were prepared by dissolving the RSF sponges in methanoic acid with a concentration of 20% by weight. RSF nanofibers were further prepared by electrospinning machine (type: YFSP-T, Yunfan (Tianjin) Instrument Co.,Ltd.). In the electrospinning process, a high voltage of 21 kV was applied to a droplet of SF solution at the tip (0.6 mm in internal diameter) of a blunt stainless steel needle (20G). The solution flow rate was controlled at 1.2 mL/h. The electrospun nanofibers were collected on a target drum which was placed at a distance of 15 cm from the needle tip and rotated at 500 rpm. After manufacturing, the RSF nanofibers were placed in a fume hood at room temperature for 48 h. Before using, the RSF nanofibers were fragmented in a cryogenic milling chamber filled with liquid nitrogen.

**Extraction of Purified PRP****.** A batch of 6-week-old rats (sixteen rats, 200-250 g of body weight) were anesthetized and about 8 ml of rat anticoagulant whole blood could be obtained. Then, the whole blood samples were centrifugated at 500 × g for 10 min and the red blood cells were separated from plasma. The upper platelets-containing plasma (about 4 mL) was transferred to another sterile tube for a second centrifugation at 800 × g for 10 min to pellet the platelets. The two-thirds of supernatant plasma was discarded and one-third of PRP (about 1.4 ml) was harvested and stored at -80 ^o^C before use.

As for CCS-RSF@PRP hydrogel preparation, CCS and RSF composite glycoprotein hydrogel (termed as CCS-RSF hydrogel) was firstly formed by simply dissolving CCS in DDW (2 wt%) and dispersing the desired amount of RSF in above solution. The gelation occurred in 1-2 minutes upon vortex. Hydrogel loaded with PRP (termed as CCS-RSF@PRP hydrogel) were fabricated by briefly mixing 10 v/v% PRP solution with CCS-RSF hydrogel and then co-incubated at 37 ^o^C for 30 min, thus 1.4 mL of PRP suffice to prepare 14 mL of artificial product CCS-RSF@PRP hydrogel for subsequent research.

**Morphology of Hydrogels.** For SEM samples preparation of CCS sponge, CCS-RSF hydrogel, CCS-RSF@PRP hydrogel and RSF nanofibers, samples were flash-frozen by liquid nitrogen, cut with a scalpel, and then lyophilized. And at least three random areas were captured for analyzing the morphology. For CCS-RSF@PRP hydrogels, the hydrogels were fixed in glutaraldehyde (2.5%, v/v) and then were washed thrice in PBS before lyophilization. The interior morphology of CCS-RSF hydrogels labeled with FITC were also investigated through CLSM.

**Rheology Properties of Hydrogels****.** The storage modulus (G’) and loss modulus (G”) were recorded over an angular frequency (ω) sweep from 0.1 to 100 rad/s under a strain (γ) of 2% and a strain sweep from 0.1 to 1000% at a frequency of 1 rad/s. Besides, the self-healing property of the hydrogels was also investigated by monitoring the changes of G’ and G” under continuous strain sweep with an alternative large oscillation force (300%) and a small one (1%). Three cycles were carried out.

**Swelling Behavior of Hydrogel.** To investigate the swelling property of CCS-RSF@PRP hydrogel, 1 mL hydrogel was immersed in PBS and artificial saliva at 37 ^o^C, the swollen volume of the hydrogel samples was recorded after carefully removing PBS and artificial saliva at 0, 2, 4, 6, 8, 12, 24 and 48 h, respectively. The swelling ratio was calculated as follows: swelling ratio = VS / V0 * 100%, where V0 was the volume of initial samples and VS was the volume of the swollen sample at different observation points.

**Degradation Behavior of Hydrogel.** To investigate the biodegradation property of CCS-RSF@PRP hydrogel, 1 mL hydrogel was immersed in PBS at 37 ^o^C, shaking at 50 rpm. The supernatant was collected after incubation for 1, 2, 3, 4, 5, 6, and 7 days, and the supernatant was freeze-dried and weighed.

**Cytocompatibility Evaluation of Hydrogels.** The cytotoxicity of hydrogels was evaluated using cell counting kit-8 assay kit (CCK-8, Solarbio, CA1201). Briefly, 5 × 10^3^ GECs and GFs, and 1 × 10^4^ PLFs and OCCM-30 cells per well were seeded into the 96-well plate with 100 μL of cell medium containing 10% FBS. After cultured for 24 hours in a 37 ^o^C humidified incubator contained 5% CO_2_, the medium was replaced with fresh medium containing 10% CCS-RSF gel, PRP or CCS-RSF@PRP gel. After incubated for 1, 2, 3 days, the medium was removed and washed by PBS. Then, cell viability was evaluated by CCK-8 assay. And the absorbance at 450 nm was measured with a microplate reader (Thermo Scientific Appliskan, USA).

The cytocompatibility of hydrogels was tested by live/dead assay kit. Briefly, GECs cells at a density of 1 × 10^5^ cells per well were seeded into the 12-well plate with 1 mL cell medium containing 10% CCS-RSF hydrogel, PRP, CCS-RSF@PRP hydrogel. After incubating for 1, 2, 3 days, GECs cells were stained by live/dead assay kit as above and observed by CLSM.

**PDGF-BB, TGF-β, VEGF,** **ATP and Ca^2+^ Release Experiment.** For PDGF-BB, TGF-β, VEGF, ATP and Ca^2+^ release studies, unactivated PRP, thrombin-activated PRP (1 mL of PRP was activated by 80 µL of the activation solution which was fabricated through mixing 2000 IU of thrombin with 4 mL of 2 wt% CaCl_2_ solution), CCS-RSF@PRP hydrogel which contained the same amount of PRP as above were incubated in 1 mL PBS (37 ^o^C, 5% CO_2_) for specific time. The supernatant was collected and the concentration of PDGF-BB, TGF-β, VEGF, ATP and Ca^2+^ released into the medium was determined by the rat PDGF-BB, TGF-β and VEGF ELISA kit (CUSABIO, CSB-E08924r, CSB-E04727r, CSB-E07352r), ATP Assay Kit (Beyotime, S0026) and Calcium Colorimetric Assay Kit (Beyotime, S1063S).

**Tube Formation Assay and** **Cells Migration.** For tube formation assay, 200 µL thawed Matrigel (BD, USA) was added into a pre-cooled 24-well plate and incubated for 30 min at 37 ^o^C. Then, HUVECs were seeded on the gel-coated plate at a density of 5 × 10^4^ cells per well. After that, the seeded HUVECs were subjected to different conditions: medium containing 10% CCS-RSF hydrogel, PRP and CCS-RSF@PRP hydrogel, respectively. After incubation for 6 h, the plates were washed with PBS and stained with Calcein-AM and observed using an optical microscope (Leica, Germany). The number of junctions was quantified using the “Image J 2.1.0/1.53c” software.

**Gingival Epithelial Cells, Gingival Fibroblasts and Endothelial Cells Migration.** The effect of hydrogels on GECs, GFs and HUVECs migration was determined using Transwell assay. First, 24-well specific plates (8.0 μm) were used for the Transwell migration assay. Briefly, GECs, GFs and HUVECs were seeded in the upper chamber of a Transwell plate at a density of 1 × 10^4^ cells per well, respectively. Fresh medium containing 10% CCS-RSF hydrogel, PRP, CCS-RSF@PRP hydrogel, respectively were placed into lower chamber and co-cultured with GECs, GFs and HUVECs for 24 hours. Eventually, the migrated cells on the bottom side of the filter were fixed with 4% paraformaldehyde and then stained with crystal violet solution (0.5%) for 30 min and then photographed and analyzed under an optical microscope. Finally, quantitative analysis of Transwell assays was conducted using “Image J 2.1.0/1.53c” software.

**ROS Scavenging Capability of CCS-RSF@PRP Hydrogel.** To evaluate the H_2_O_2_ scavenging effect of CCS-RSF@PRP hydrogel, DCFH-DA was used as a sensitive probe for H_2_O_2_. 10 μL of hydrogel samples (CCS-RSF@PRP hydrogels with different CCS concentration) were immersed in 90 μL of H_2_O_2_ solution (20 mM). After incubation for 30 min, 1 μL of DCFH-DA working solution (10 μM) was added and incubated for further 30 min. The maximum fluorescence intensity at 515 nm was measured using a microplate reader with 488 nm as excitation light. The scavenging effect of H_2_O_2_ was calculated by the following formula: H_2_O_2_ scavenging %=(Ib-Ih)/Ib×100%, where Ib and Ih were the fluorescence intensity of the blank (H_2_O_2_+DCFH-DA) and the hydrogel sample, respectively.

To test the scavenging effect on DPPH radicals, 20 μL of hydrogel samples with different CCS concentrations were immersed in 2 mL of DPPH solution (0.1 mM, in 95 wt% ethanol) for 30 min in dark. The UV absorptance spectrum was scanned between 400 and 800 nm at 1 nm intervals, and the scavenging effect on DPPH radicals was calculated by the following formula: DPPH scavenging %= (Ab-Ah)/Ab×100%, where Ab and Ah were the maximum absorptance of the blank and the hydrogel samples, respectively.

The scavenging ability of CCS-RSF@PRP hydrogel on superoxide anion and hydroxyl free radical was tested according to specification of Superoxide Anion Scavenging Capacity Assay Kit (Solarbio, BC1415) and Hydroxyl Free Radical Scavenging Capacity Assay Kit (Solarbio, BC1320).

A reactive oxygen species assay kit (2,7- dichlorodihydrofluorescein diacetate, DCFH-DA; S0033S, Beyotime, China) was used to test the cellular ROS scavenging activity. Firstly, the GECs cells (1 × 10^4^ per well) were seeded on confocal microscopic dish and cultured for 24 h. Then, GECs were treated with Rosup reagent (50 μg/mL) in reactive oxygen species assay kit for 30 min, and then fresh media containing CCS-RSF@PRP hydrogel was added. After incubation for 30 min, the cells were washed three times and DCFH-DA probe molecule (10 μM) was added under dark condition and incubated at 37 ^o^C for 30 min. The ROS levels were measured using CLSM and flow cytometry (C6, BD, USA). The group without Rosup stimulation was used as a control.

To explore the effects of H_2_O_2_ to the cell viability of GECs, 5 × 10^3^ GECs per well were seeded into the 96-well plate with 100 μL of cell medium containing 10% FBS. After cultured for 24 h in a 37 ^o^C humidified incubator contained 5% CO_2_, the medium was replaced with fresh medium containing 10% H_2_O_2_ (20 mM) and CCS-RSF@PRP hydrogel. After incubated for 1 day, the medium was removed and washed by PBS. Then, cell viability was evaluated by CCK-8 assay. And the absorbance at 450 nm was measured with a microplate reader (Thermo Scientific Appliskan, USA). In addition, the effect of H_2_O_2_ to cells apoptotic was evaluated using Annexin V-FITC apoptosis detection kit through flow cytometry (C6, BD, USA). 5 × 10^5^ GECs per well were seeded into the 6-well plate with 100 μL of cell medium containing 10% FBS. After cultured for 24 h, the medium was replaced with fresh medium containing 10% H_2_O_2_ (20 mM) and CCS-RSF@PRP hydrogel. After incubated for 2 days, GECs cells were subjected according to specification of Annexin V-FITC apoptosis detection kit. Thereinto, Annexin V-FITC probe with green fluorescence was used to detect the efflux of phosphatidylserine, a characteristic of apoptosis, and the red fluorescent propidium iodide (PI) was used to stain late apoptosis and necrotic cells that lost cell membrane integrity.

**LPS and Cytokines Scavenging Studies****.** To quantify the effect of LPS neutralization by PRP, unactivated PRP and activated-PRP isolated from CCS-RSF@PRP hydrogel (40 mg/mL) were mixed with LPS from *E. coli* K12 (Invivogen) with varied amounts of 0.1, 0.25, 0.5, and 1.0 EU/mL, respectively. For preparation of activated-PRP isolated from CCS-RSF@PRP hydrogel, after incubating CCS-RSF with PRP solution and before gel formation, the mixture was centrifuged at 1000 rpm for 2 minutes. Then the CCS-RSF precipitate was removed, and activated PRP was isolated. The mixtures were incubated for 30 min and then spun down at 500 g for 10 min to pellet the PRP. The free LPS content in the supernatant was quantified by using Limulus amebocyte lysate (LAL) assay (Thermo Fisher Scientific). All experiments were performed in triplicate.

To evaluate cytokines scavenging with activated-PRP, including TNF-α, IFN-γ, and IL-1β, 100 μL of activated-PRP samples (10, 40 mg/mL) mixed with TNF-α (250 pg/mL), IFN-γ (5 pg/mL), and IL-1β (1.5 ng/mL) in PBS were incubated at 37 ^o^C for 30 min. Following the incubation, the samples were centrifuged at 500 g for 10 min to pellet the PRP. Cytokine concentrations in the supernatant were quantified by using ELISA (CUSABIO). All experiments were performed in triplicate.

**Coculture System between BMDMs and GECs.**BMDMs stimulated by M-CSF for 6 days were seeded in 6-well plates with a density of 2 × 10^5^ per well. The culture medium was supplied with LPS-*P. g*, LPS+CCS-RSF, LPS+PRP and LPS+CCS-RSF@PRP hydrogel. 1.5 × 10^5^ GECs were seeded on the inserted chamber with a pore size 8 μm (Corning, 3450). After coculturing for 2 days, the migrated GECs on the bottom side of the filter were fixed with 4% paraformaldehyde and then stained with crystal violet solution (0.5%) for 30 min and then photographed and analyzed under an optical microscope. Finally, quantitative analysis of Transwell assays was conducted using “Image J 2.1.0/1.53c” software.

**Macrophage Activation by Hydrogels.** To investigate the phenotypic shift of M1 macrophages in CCS-RSF@PRP hydrogel, M1 type macrophages were obtained by treating BMDMs with 40 ng/mL LPS for 48 h. Then, M1 macrophages were further treated with PBS, IL-4 (40 ng/mL), or CCS-RSF@PRP hydrogel for another 48 h. After incubation, cells were collected by centrifugation and washed twice with cold PBS. Collected cells were stained with PE-labeled F4/80 antibody, FITC-labeled anti-MHC II antibody, and APC-labeled CD206 antibody and analyzed by flow cytometry.

**Real-Time Quantitative Polymerase Chain Reaction (RT-qPCR).** M2 macrophages-related gene expression was detected by RT-qPCR. Macrophages were treated with different medium as described above for 48 h. Total RNA was isolated and extracted using HR Total RNA kit (OMEGA, R6812-02). The concentration was determined from spectrophotometric optical density measurement (Nanodrop2000, Thermo Fisher Scientific, USA). The OD260/OD280 ratio in all samples ranged from 1.7 to 2.0 to ensure RNA purity. First-strand cDNA was synthesized by reverse transcription method according to the protocol provided by the GoScript Reverse Transcription System (Promega, A5001). Three main genes closely related to M2 macrophages, including CD206, Arg-1, VEGF-A were analyzed in this study. RT-qPCR was conducted on an IQ5 detection system (Bio-Rad, USA) using SYBR Green Master Mix (Promega, A6001) and a thermal cycling was performed at 95 ^o^C for 30 s, followed by 45 cycles at 95 ^o^C for 10 s and 60 ^o^C for 30 s. All mRNA expression levels were normalized to the β-actin housekeeping gene. The forward and reverse primer sequences were listed in Table S1.

**Western Blot.** RIPA lysis buffer (Beyotime, P0013B) containing 1 mm PMSF (Beyotime, ST506) was applied to obtain total cell lysates of platelets, macrophages and periodontium, and then the cells and periodontium were incubated on ice for 30 min, respectively. The lysed samples were centrifuged at 12000 × g for 5 min to extract the supernatant and the total protein concentration was quantified by BCA protein assay kit (Beyotime, P0012S). After mixing with loading buffer containing bromophenol blue, protein samples were denatured on 100 ^o^C for 10 min. Then protein samples were separated by a 10% polyacrylamide gel and transferred onto PVDF membrane (0.45 µm). The membrane was blocked with 5% skimmed milk (w/v) powder at 37 ^o^C for 30 min and then incubated with rabbit polyclonal antibody to Arg-1 (1:1000, Abcam, ab233548), iNOS (1:1000, Abcam, ab210823), p-Erk1/2 (1:1000, Abcam, ab17942), p-MEK1 (1:1000, Abcam, ab96379), p-IκBα (1:1000, Cell signaling technology, 2859T), p-NF-κB (1:1000, Cell signaling technology, 3033T), TNF-α (1:1000, Cell signaling technology, 4814T), CD40 (1:1000, ABclonal, A13285), CD62P (1:1000, ABclonal, A1425), CD121b (1:1000, Bioss, bs-2595R) and β-actin (1:1000, Abcam, ab8227) overnight at 4 ^o^C. The membrane was washed with TBST for five times and incubated with a goat anti-rabbit horseradish peroxidase-conjugated secondary antibody for 2 h at room temperature. Finally, protein bands were captured by Chemiluminescence Imaging system (ChemiScope 6000 Pro, China) using the enhanced chemiluminescence (ECL) western blotting substrate. The signal intensity of immunoreactive bands were quantified by ImageJ software and normalized using β-actin.

**Residence Behavior of CCS-RSF@PRP Hydrogel *in vivo*.** The *in vivo* residence of CCS-RSF@PRP hydrogel was investigated by non-invasive fluorescence imaging. Cy5-NHS was coupled to CCS-RSF@PRP hydrogels through amidation reaction. Then, Cy5-labeled CCS-RSF@PRP hydrogels were injected into the lesion location of periodontal pocket. Fluorescence images were recorded and the fluorescence intensity was quantified at schedule time points using the Maestro imaging system (CRI, USA). After treatment, the biotoxicity of CCS-RSF@PRP hydrogels to the heart, liver, spleen, lungs and kidneys was examined through H&E staining. In addition, CCS-RSF@PRP hydrogels were injected under the oral mucosa to test its biosafety through H&E staining.

***In vivo* Experiment.** The alveolar bone, molars, and gum tissues on the left side of the upper jaw were harvested and scanned by a Micro-computational tomography (Micro-CT) at 50 kV. After 3D reconstruction (RadiAnt DICOM Viewer), the vertical distance between the cemento-enamel junction (CEJ) and the alveolar bone crest (ABC), namely CEJ-ABC, which represents the degree of alveolar bone loss, was measured. In addition, the parameters of diastema distance of maxillary first with second molars and second with third molars were measured and analyzed for the evaluation of tissue destroy. The periodontal tissue was isolated, homogenized in ice lysis buffer with a ball mill, and then centrifuged at 12,000 × g at 4 ^o^C for 10 min. The resulting supernatants were collected for quantification of the levels of LPS, TNF-α, and IL-1β by ELISA kit and M1, M2 macrophages-related gene expression, including CD206, VEGF-A, TGF-β, IL-10, TNF-α and CD86 by RT-qPCR.

**Histological Analysis, Immunofluorescence and Immunohistochemical Staining.** The tissue samples were decalcified in 10% ethylene diamine tetraacetic acid (EDTA) for one months, with EDTA solution refreshed once a week. Then, the samples were dehydrated in a graded series of ethanol, embedded in paraffin, and sectioned at a thickness of 6.0 μm for hematoxylin–eosin (H&E) staining (Solarbio, G1120), Masson’s trichrome staining (Solarbio, G1340) and Sirius Red staining (Solarbio, G1472). The inflammatory response and the histology of the periodontium were observed by microscope.

In addition, immunofluorescence staining for CD68 and CD206 was performed to detect the M2 macrophages level of the periodontal tissue. The periodontal tissue sections were washed with PBS, and then blocked with goat serum at room temperature. Then sections were incubated with the primary antibody of a mouse polyclonal antibody to CD68 (1:200, Abcam, ab955), a rabbit polyclonal antibody to CD206 (1:400, Cell Signaling Technology, 24595). After incubation for 3 h, sections were washed with PBS, and then incubated with Alexa Flour 488 goat anti-rabbit IgG (1:500, Invitrogen, A11034) and Alexa Flour 594 goat anti-mouse IgG (1:500, Invitrogen, A11032) corresponding to the primary antibody. After being mounted with DAPI containing mounting medium, images were obtained by CLSM and quantitatively analyzed by ImageJ software. Immunofluorescence staining for DHE (Beyotime, S0063) and RUNX2 (Beyotime, AF2593) were conducted to evaluate ROS level and bone regeneration of the periodontal tissue following a standard protocol as reported in our previous work. Immunohistochemical staining for mouse monoclonal TNF-α (1:1000, Abcam, ab220210) was performed to evaluate the inflammation level of the periodontal tissue.


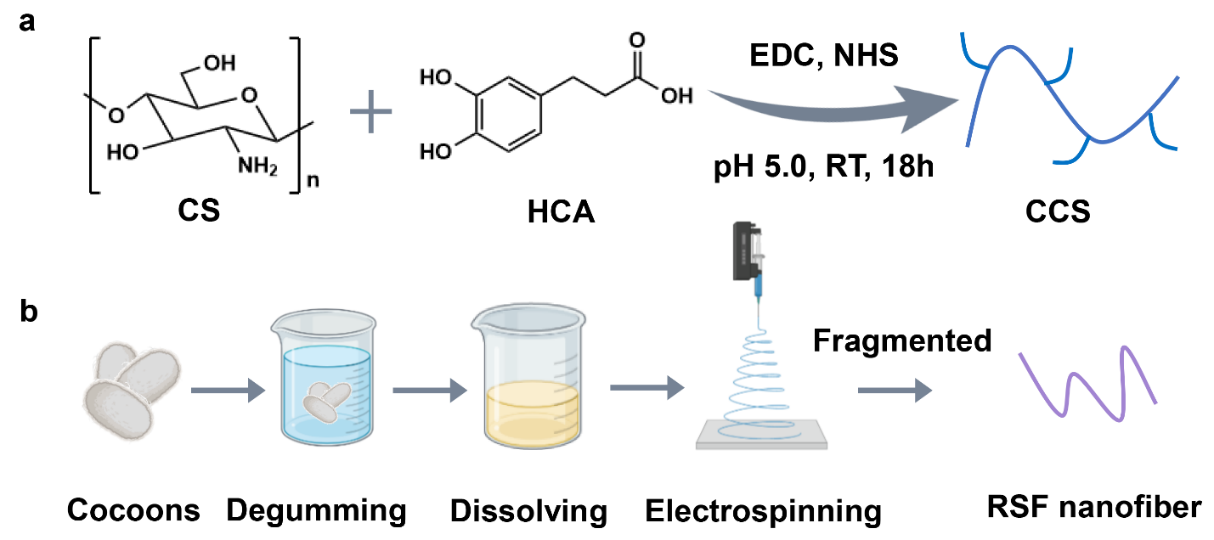


**Figure S1.** Schematic diagram of a) CCS synthesis and b) RSF nanofiber synthesis.


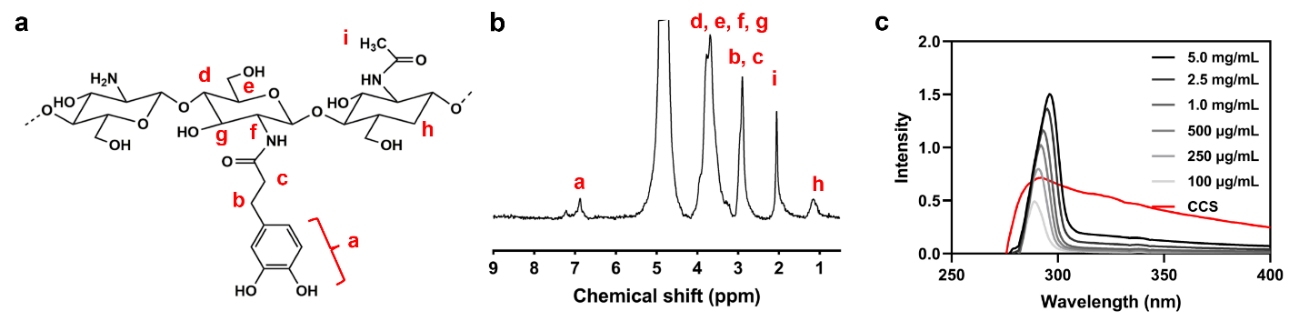


**Figure S2.** a) Structural formula of CCS. b) ^1^HNMR spectra of CCS. c) UV-vis spectra of CCS aqueous solution and HCA aqueous solution with concentration of 100, 250, 500 μg/mL and 1.0, 2.5, 5.0 mg/mL.


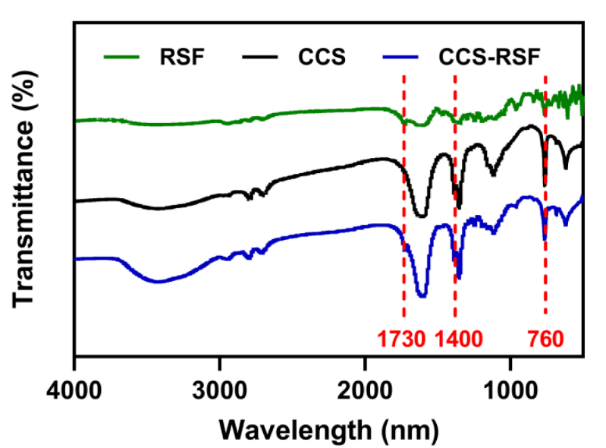


**Figure S3.** The FTIR spectrum of RSF nanofiber, CCS and CCS-RSF hydrogels after lyophilization.


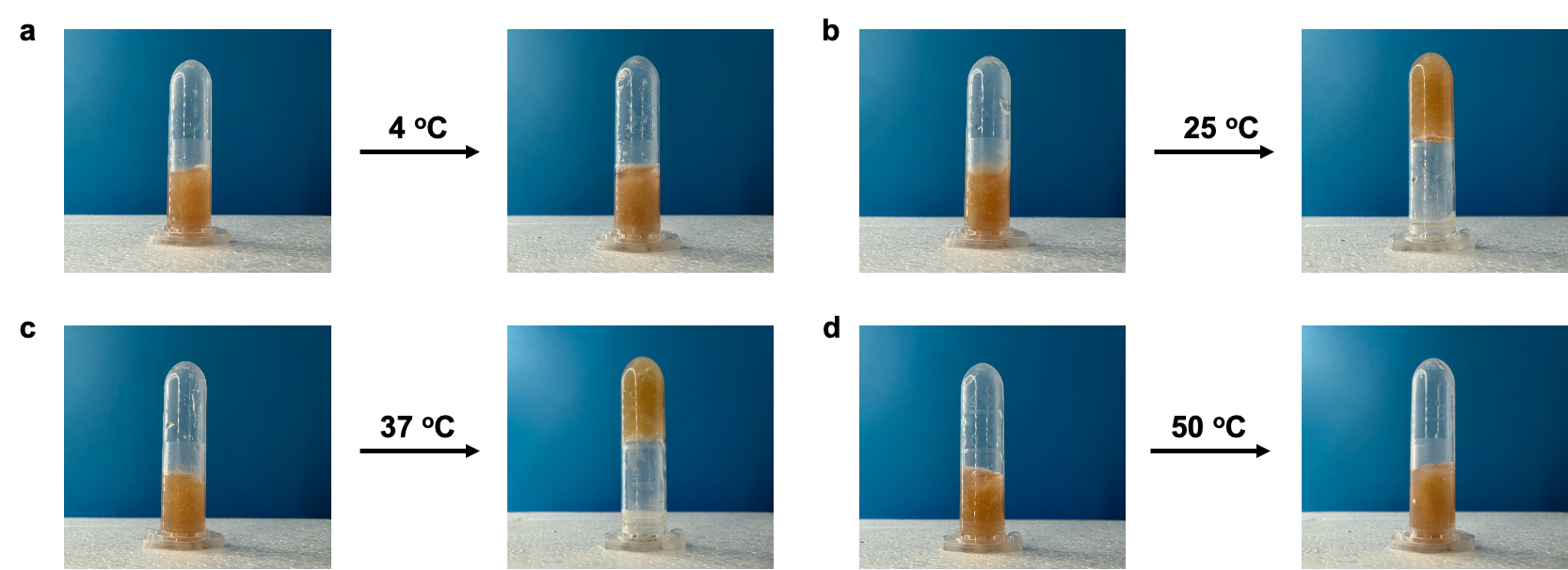


**Figure S4.** The physical photos of hydrogels synthesized at different temperatures (4, 25, 37 and 50 ^o^C)


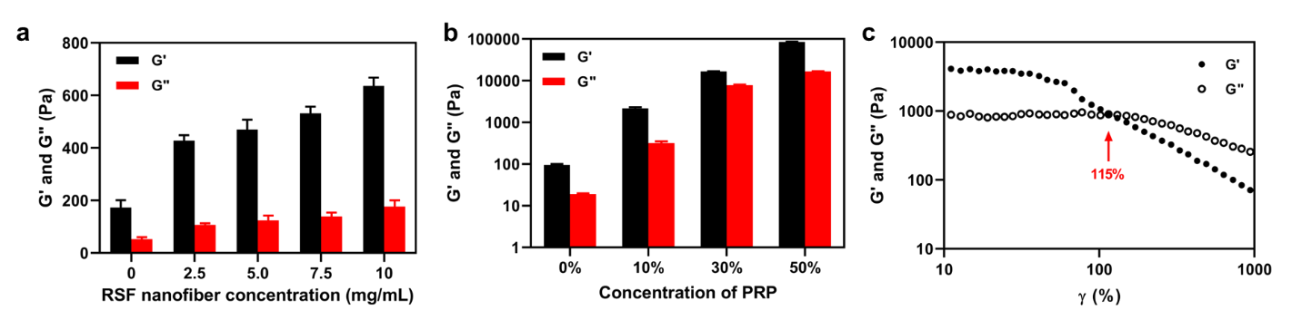


**Figure S5.** The influence of concentration of a) RSF nanofiber and b) PRP (v/v) on the modulus of CCS-RSF@PRP hydrogels. c) Rheological analysis of CCS-RSF@PRP hydrogels as a function of shear strain.

**
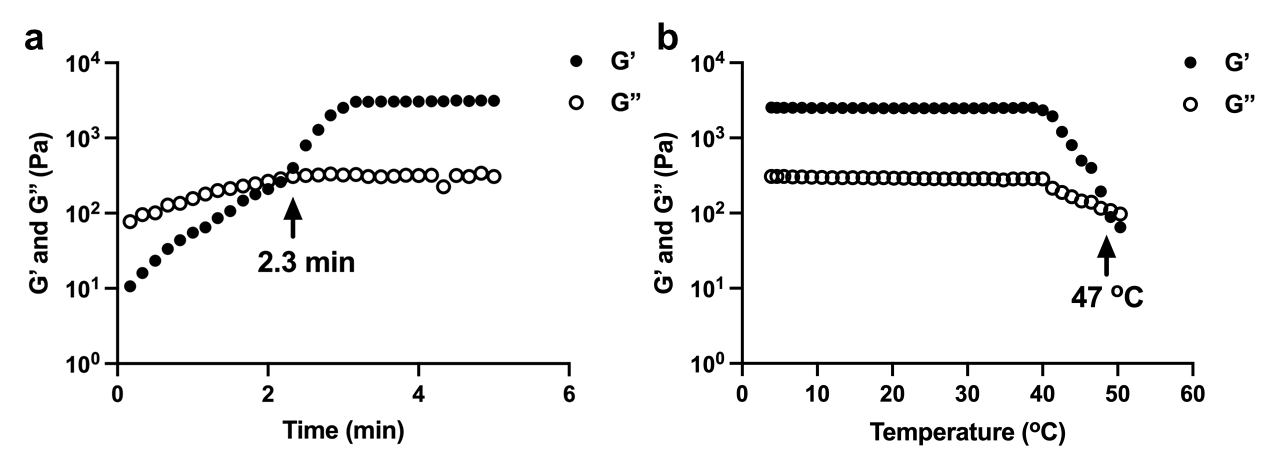
**

**Figure S6.** a) Time-sweep rheological analysis of CCS-RSF@PRP hydrogel at 37 ^o^C. b) Temperature-sweep rheological analysis of CCS-RSF@PRP hydrogel within 4-50 ^o^C.


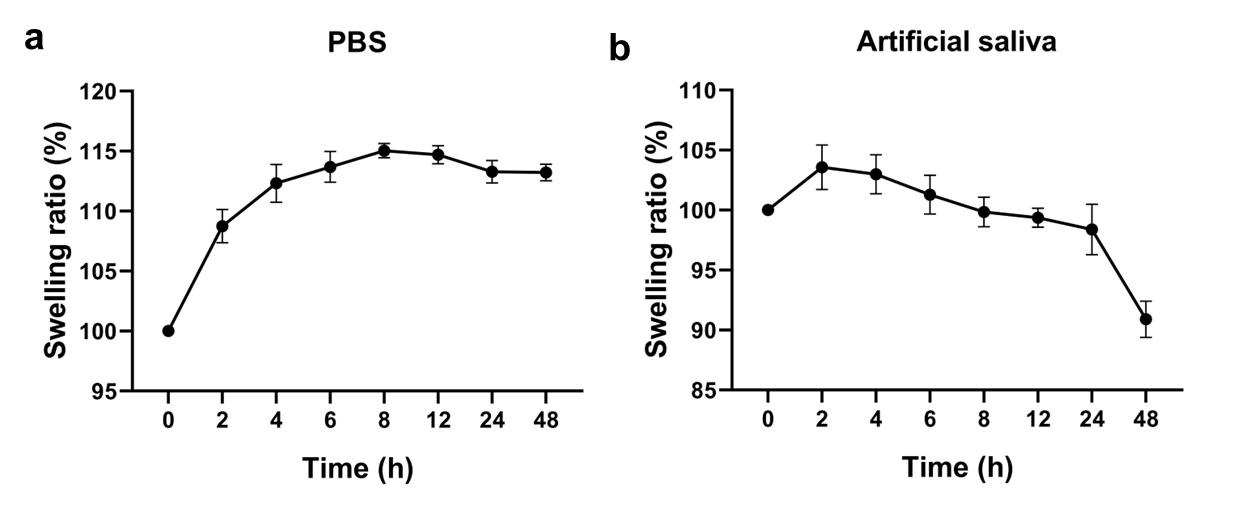


**Figure S7.** Swelling ratio curves for CCS-RSF@PRP hydrogel in a) PBS and b) artificial saliva at 37 ^o^C (n=6).

**
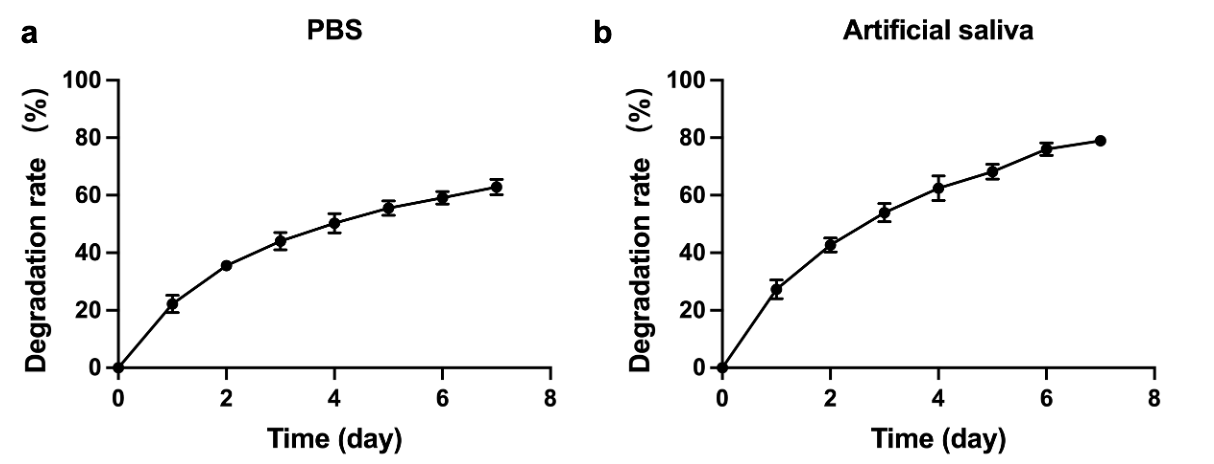
**

**Figure S8.** Degradation rate curves for CCS-RSF@PRP hydrogel in a) PBS and b) artificial saliva at 37 ^o^C (n=6).

**
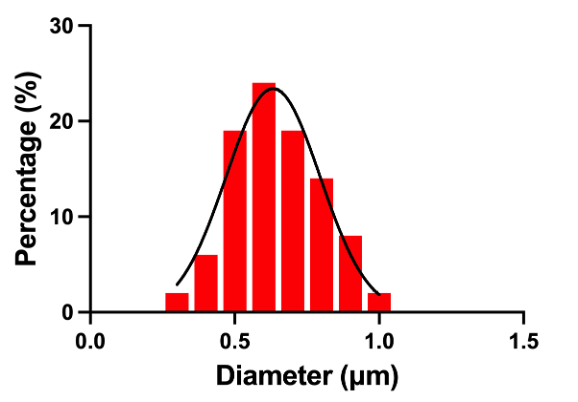
**

**Figure S9.** Diameter distribution of RSF nanofibers.


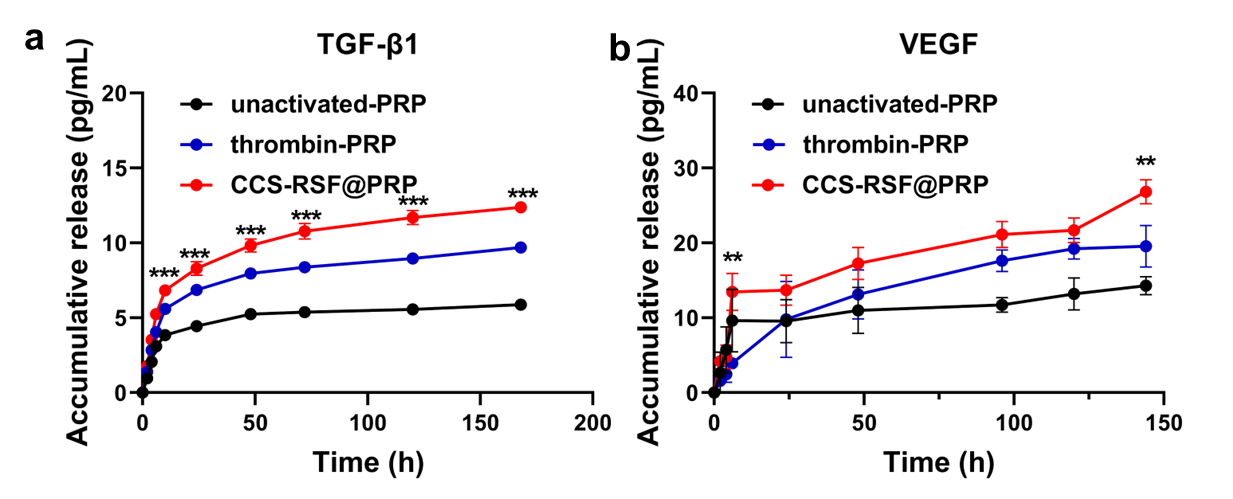


**Figure S10.** The accumulative release of a) TGF-β1 and b) VEGF from unactivated PRP, thrombin activated-PRP and CCS-RSF@PRP hydrogel (n=3).


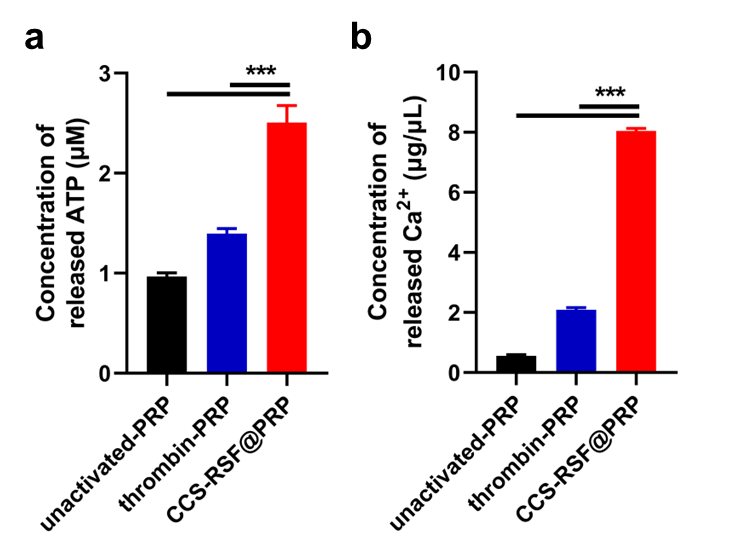


**Figure S11.** a) ATP and b) Ca^2+^ releases from activated platelets in unactivated PRP, thrombin activated-PRP and CCS-RSF@PRP hydrogel (n=3).


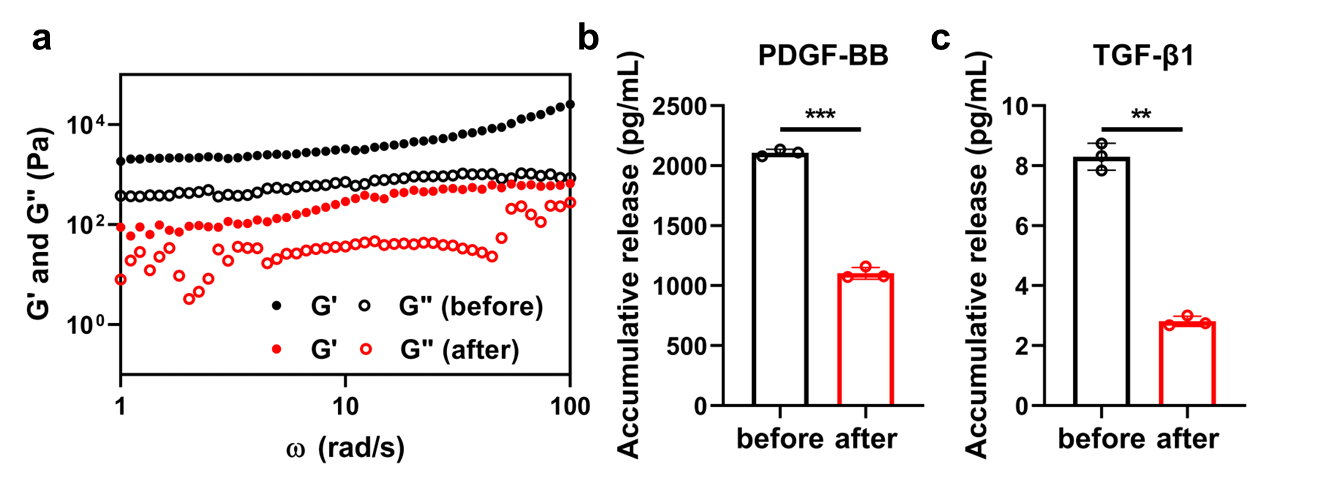


**Figure S12.** a) Storage modulus (G′) and loss modulus (G″) of CCS-RSF@PRP hydrogel after storage at 4 ^o^C for one month. b) PDGF-BB and c) TGF-β1 release from CCS-RSF@PRP hydrogel after storage at 4 ^o^C for one month (n=3).


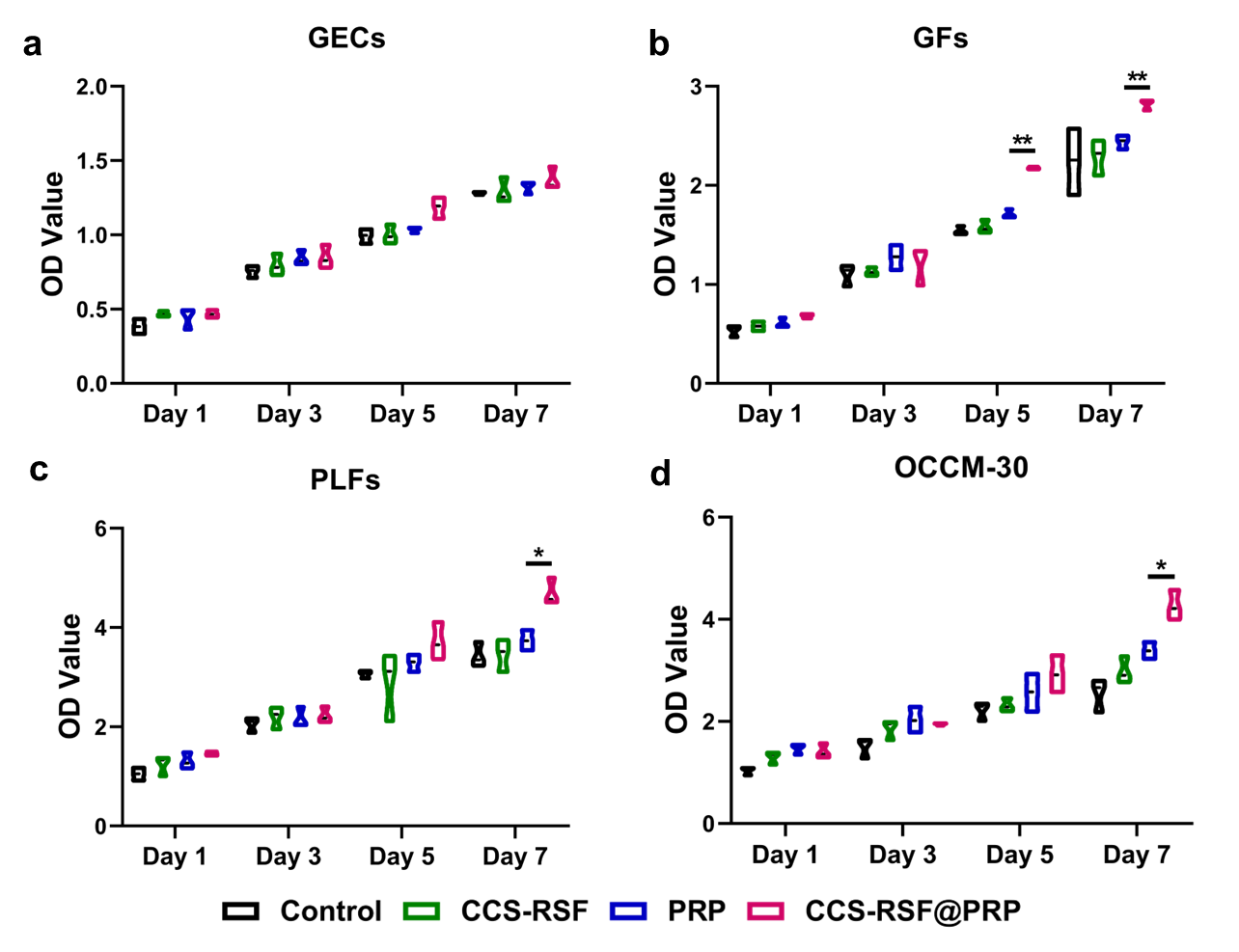


**Figure S13.** Cell proliferation of a) GECs, b) GFs, c) PLFs and d) OCCM-30 cultured with different medium for 1, 3, 5 and 7 days evaluated by CCK-8 assay (n=3).


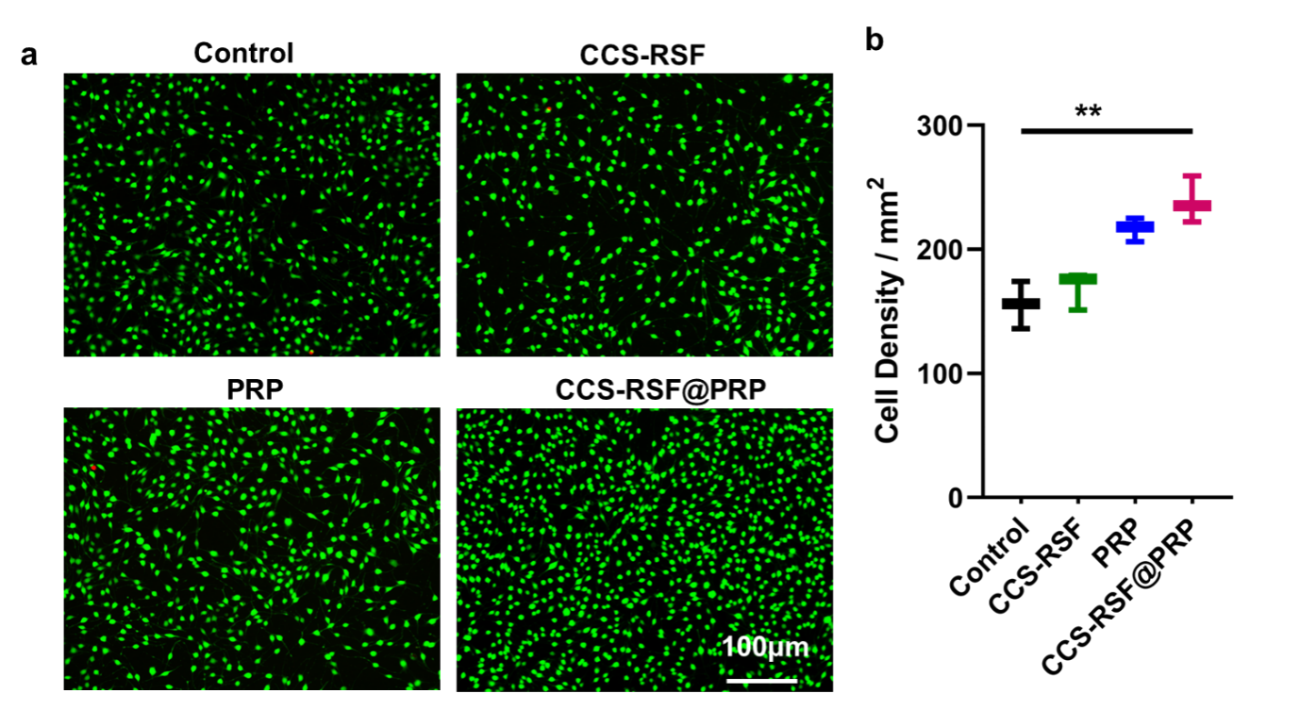


**Figure S14.** a, b) Representative fluorescence images and quantitative analysis of live/dead assay for GECs cells (n=3).


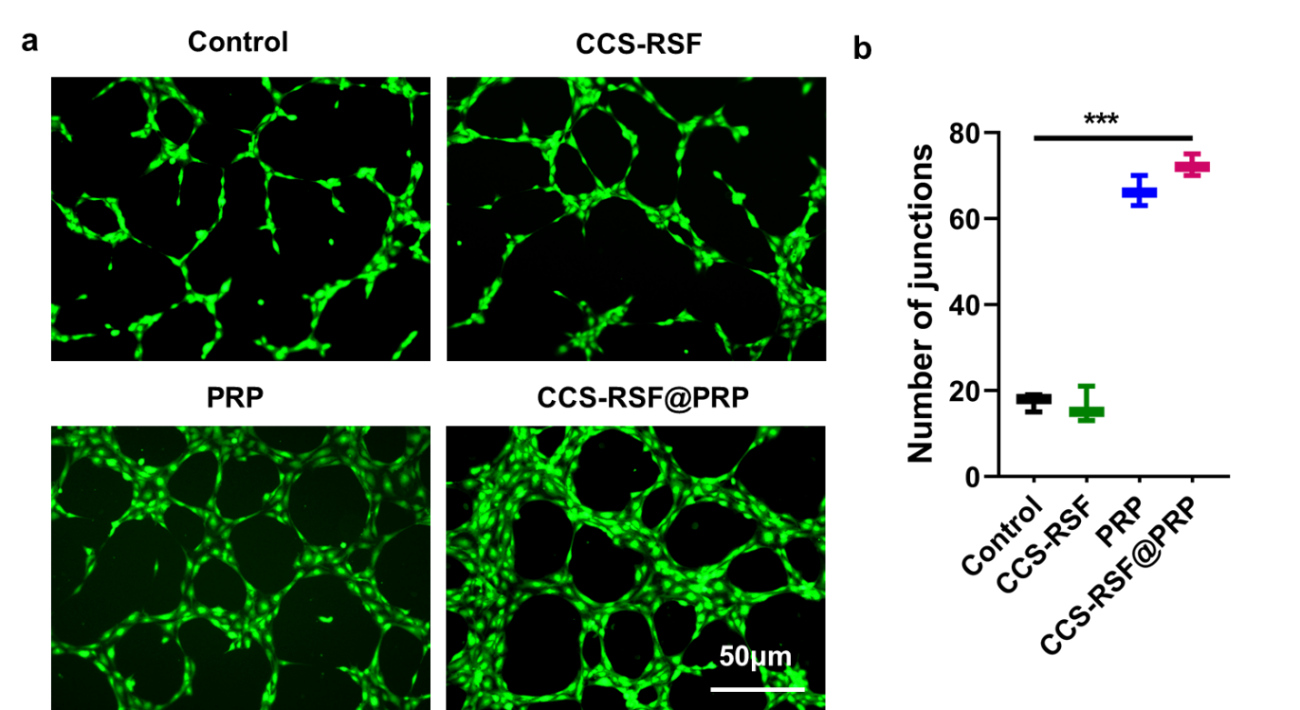


**Figure S15.** a, b) Representative images and quantitative analysis of tube formation assay for HUVECs (n=3).


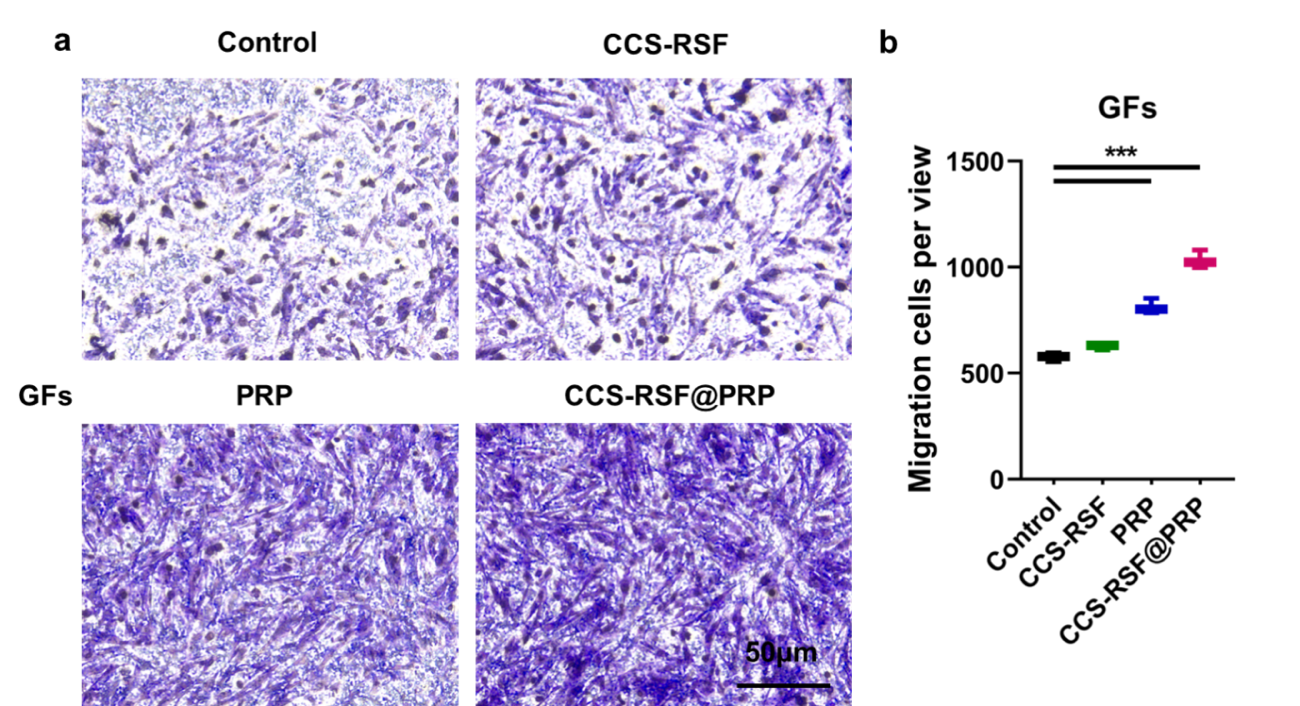


**Figure S16.** a, b) Representative images and quantitative analysis of Transwell migration assay for GFs (n=3).


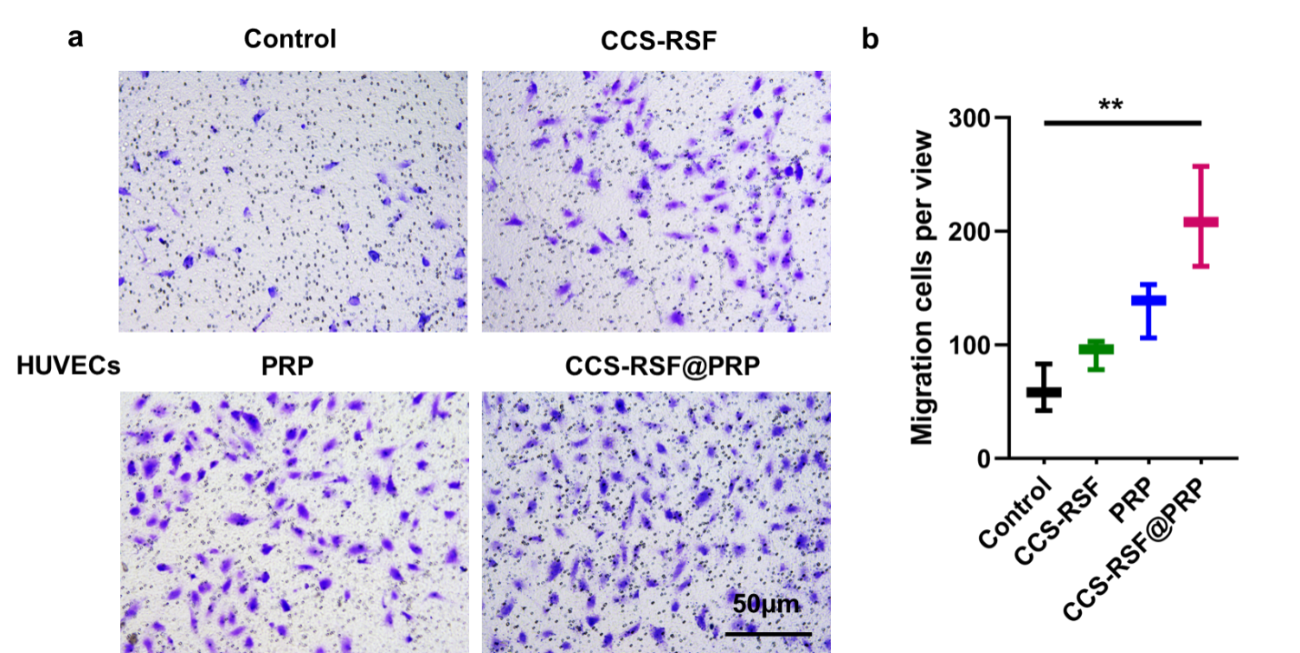


**Figure S17.** a, b) Representative images and quantitative analysis of Transwell migration assay for HUVECs (n=3).


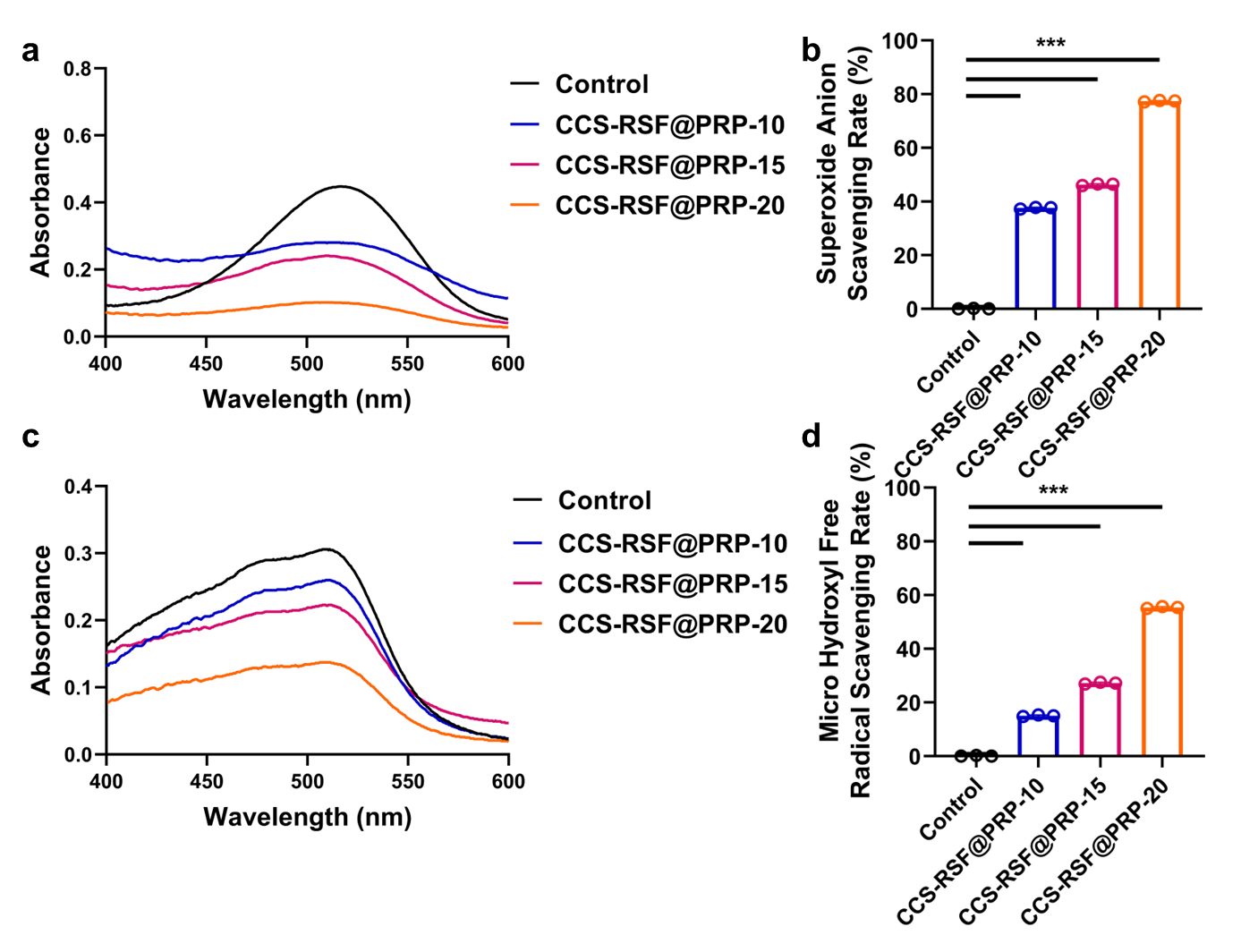


**Figure S18.** a) UV-vis spectra of superoxide anion detection. b) Superoxide anion scavenging rate of CCS-RSF@PRP hydrogel with CCS concentration of 10, 15, and 20 mg/mL, respectively (n=3). c) UV-vis spectra of hydroxyl free radical detection. d) Hydroxyl free radical scavenging rate of CCS-RSF@PRP hydrogel with CCS concentration of 10, 15, and 20 mg/mL, respectively (n=3).


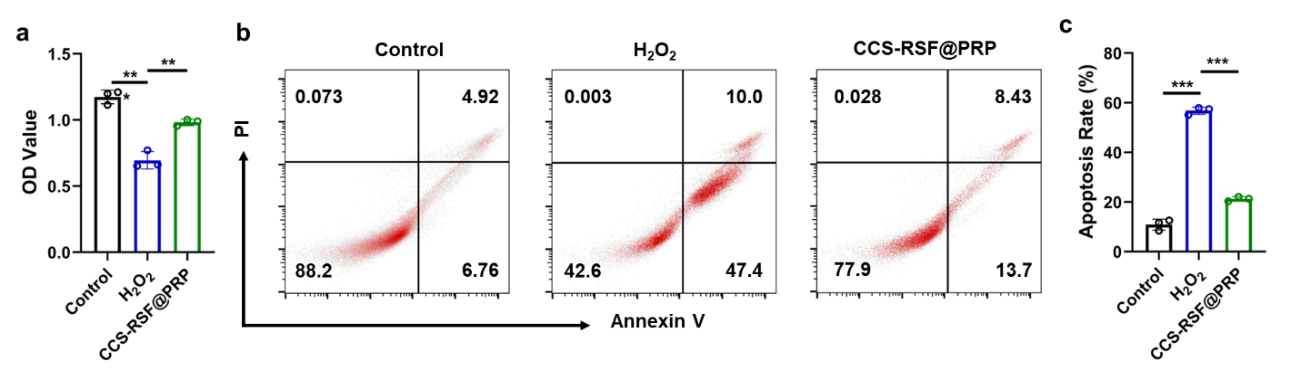


**Figure S19.** a) Cell proliferation of GECs cultured with H_2_O_2_ and H_2_O_2_+CCS-RSF@PRP hydrogel evaluated by CCK-8 assay (n=3). b) Flow cytometry analysis of PI and Annexin V of GECs for apoptosis detection. c) Apoptosis rate of GECs after treatment with H_2_O_2_ and H_2_O_2_+CCS-RSF@PRP hydrogel (n=3).


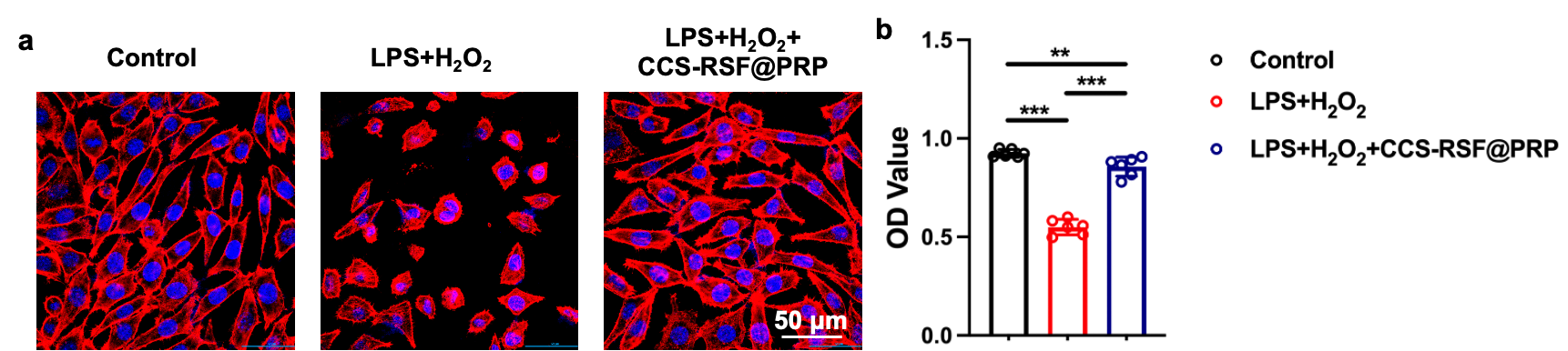


**Figure S20.** a) Representative CLSM images of GECs treated by LPS+H_2_O_2_ and LPS+H_2_O_2_ with CCS-RSF@PRP hydrogel (red: F-actin; blue: cell nuclear). b) Cell viability of GECs cultured with LPS+H_2_O_2_ and LPS+H_2_O_2_ with CCS-RSF@PRP hydrogel evaluated by CCK-8 assay (n=6).


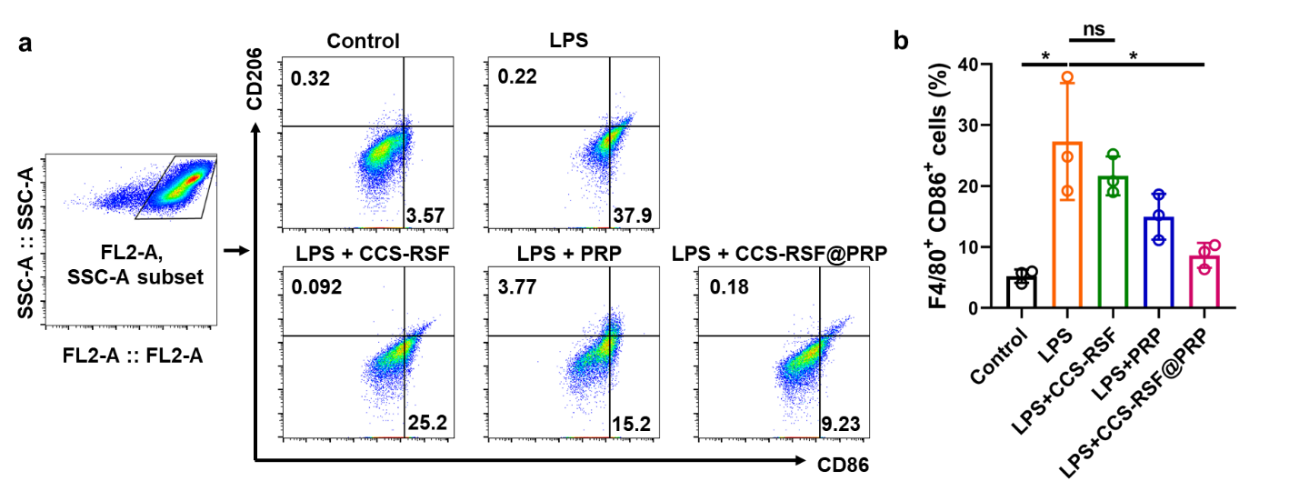


**Figure S21.** a) Flow cytometry analysis of CD206 and CD86 expression (gated on F4/80^+^ cells) of BMDMs for LPS neutralization. b) Percentage of M1 (F4/80^+^CD86^+^) type macrophages (n=3).


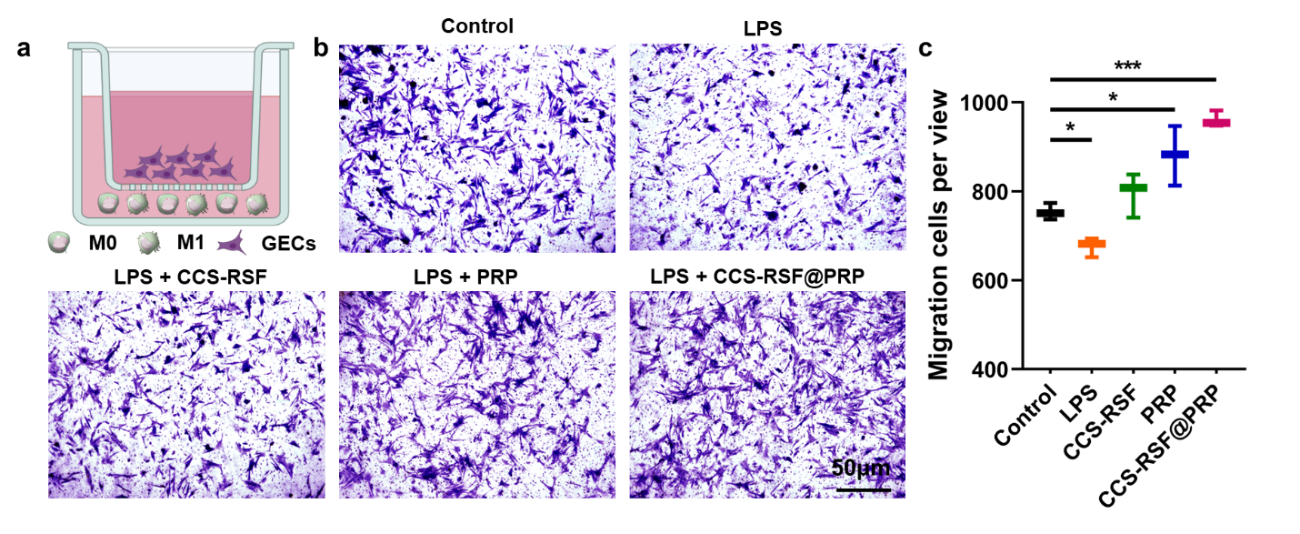


**Figure S22.** a) A coculture system was established by seeding BMDMs (M0 and M1) on the bottom and GECs on the inserted chamber. b, c) Representative images and quantitative analysis of Transwell migration assay for GECs coculturing with BMDMs treated by different mediums (n=3).


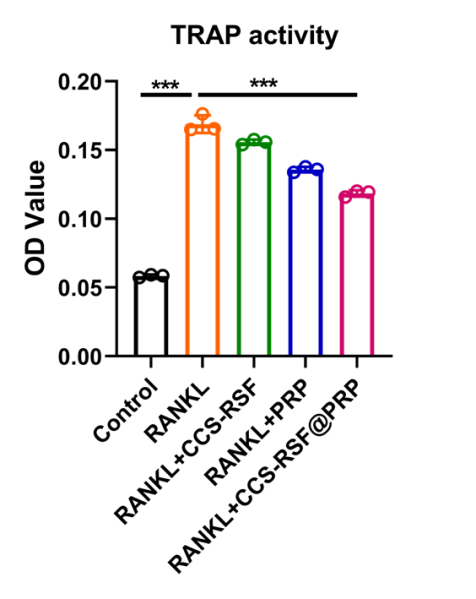


**Figure S23.** TRAP activity analysis of RAW 264.7 treated with RANKL, RANKL+CCS-RSF, RANKL+ PRP and RANKL+CCS-RSF@PRP, respectively (n=3).

**
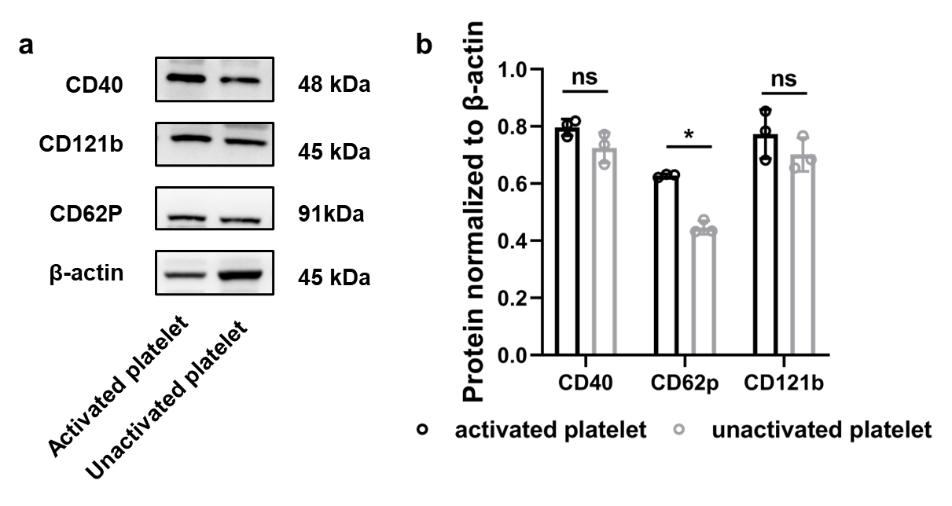
**

**Figure S24.** a, b) Protein expression level of CD40, CD121b and CD62P in activated platelet isolated from CCS-RSF@PRP and unactivated platelet group determined by western blot (n=3).


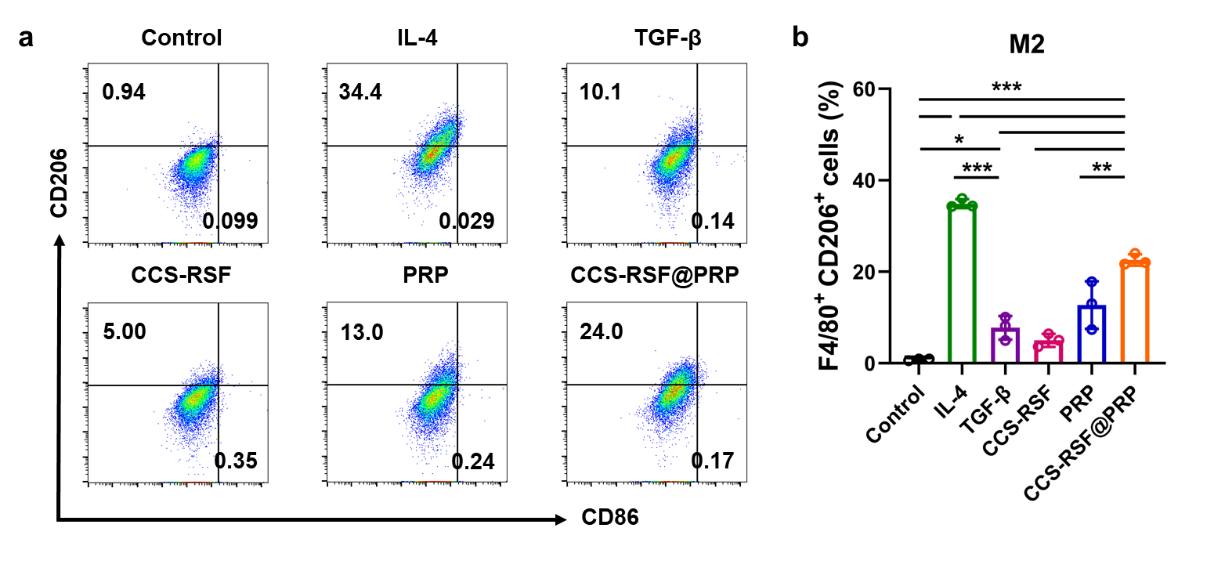


**Figure S25.** a) Flow cytometry analysis of CD206 and CD86 expression (gated on F4/80^+^ cells). Percentage of b) M2 (F4/80^+^CD206^+^) type macrophages (n=3).


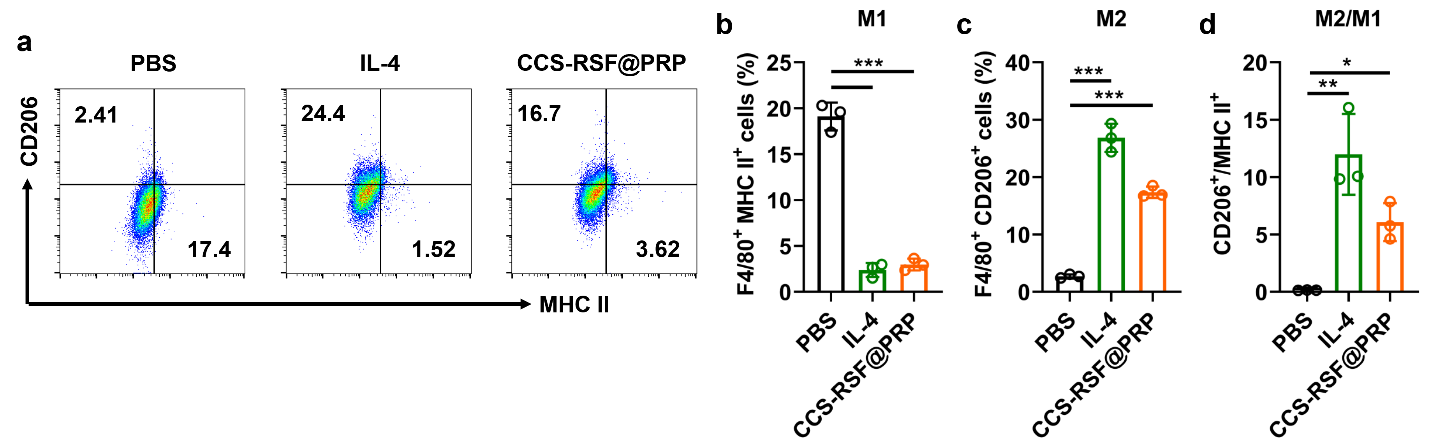


**Figure S26.** a) Flow cytometry analysis of CD206 and MHC II expression of BMDMs (gated on F4/80^+^ cells). Percentage of b) M1 (F4/80^+^MHC II^+^), c) M2 (F4/80^+^CD206^+^) and d) ratio of M2 to M1 type macrophages (n=3).


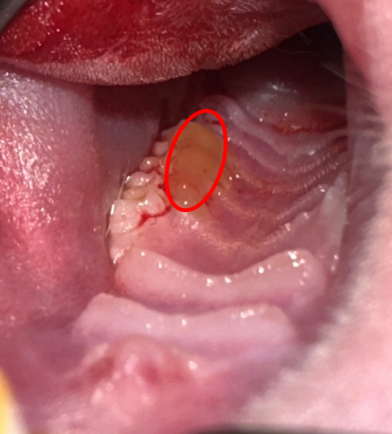


**Figure S27.** Picture of CCS-RSF@PRP hydrogel in situ curing in the periodontal pockets and onto the damaged periodontium.


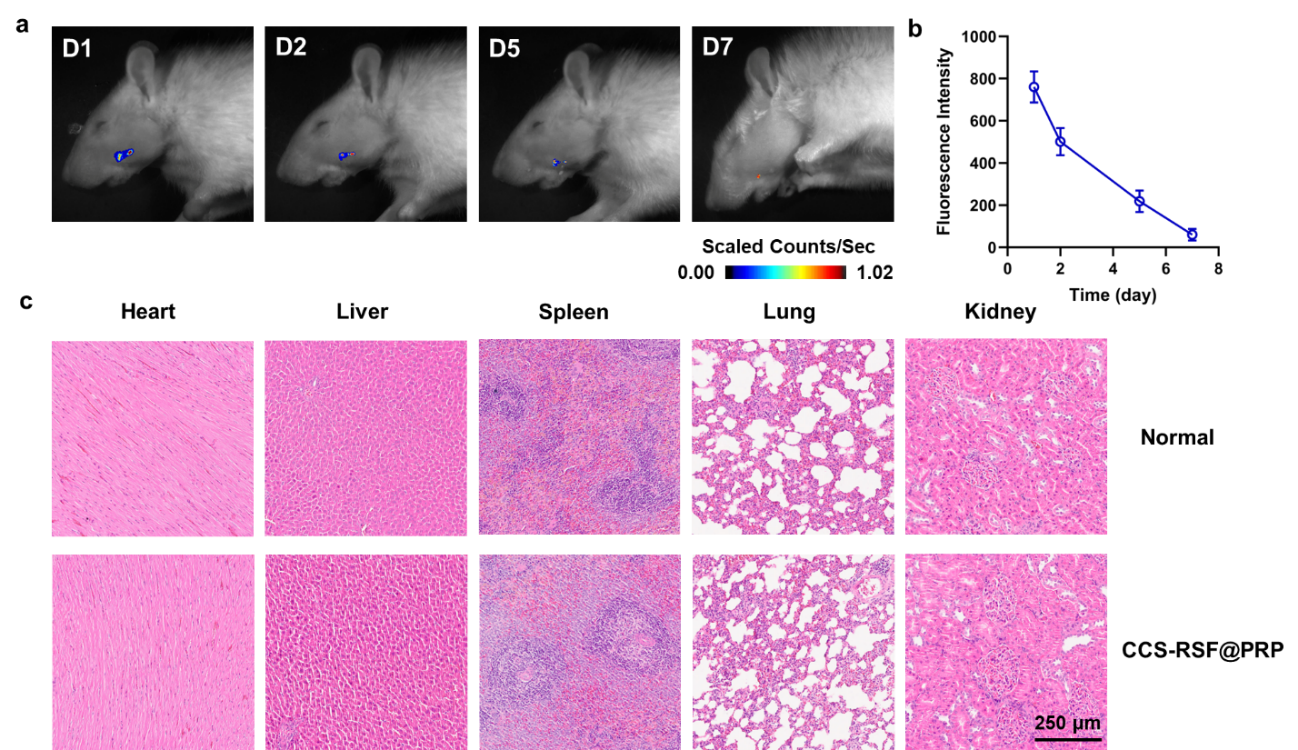


**Figure S28.** a) Time dependent fluorescent images of rats after injection with Cy5 labeled CCS-RSF@PRP hydrogels at the lesion location of periodontitis. b) The fluorescence intensity of Cy5 at different time points after injection (n=3). c) H&E staining to detect toxicity in the heart, liver, spleen, lungs and kidneys after treatment with CCS-RSF@PRP hydrogels.


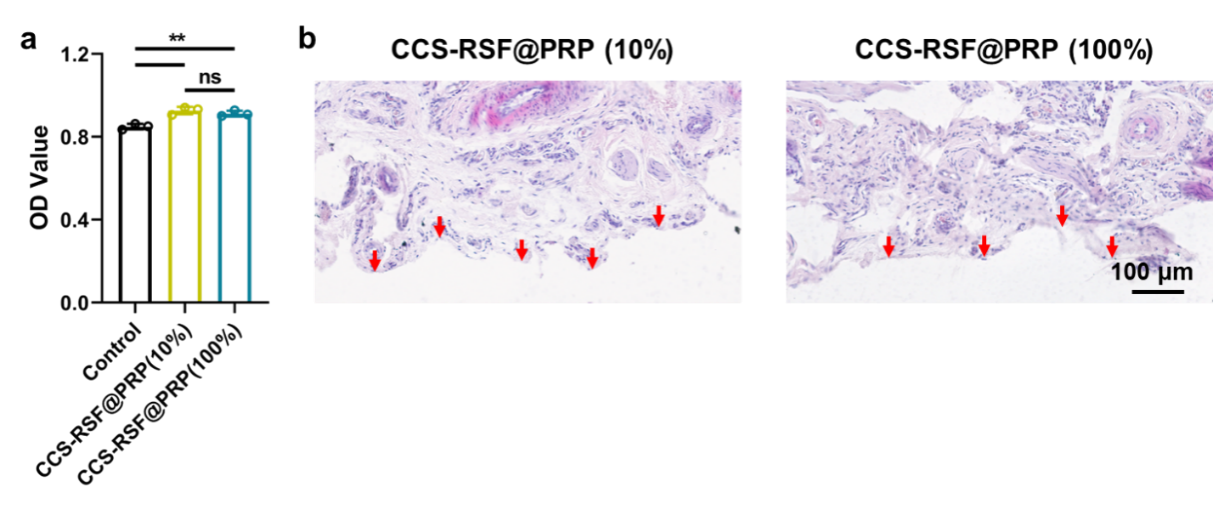


**Figure S29.** a) Cell proliferation of GECs cultured with CCS-RSF@PRP hydrogel with 10 v/v% and 100 v/v% of PRP solution evaluated by CCK-8 assay (n=3). b) H&E staining of the oral mucosa after injection of CCS-RSF@PRP hydrogels (10 v/v% and 100 v/v% of PRP solution). The red arrow represents the injection site of the hydrogel.


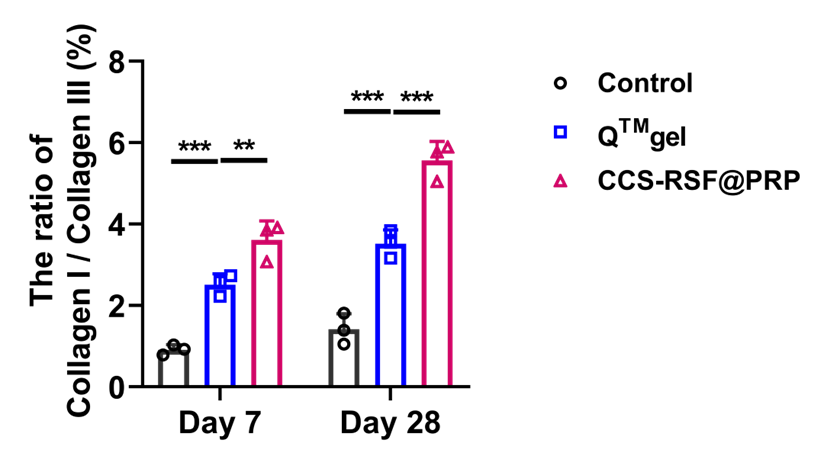


**Figure S30.** Statistical data of the ratio of Collagen I/Collagen III (%) (n=3).

Table S1 The sequences specific primers for these genes.

| Gene | Forward Sequence (5'to3') | Reverse Sequence (5'to3') |
| --- | --- | --- |
| β-actin | TCTGTGTGGATTGGTGGCTCTA | CTGCTTGCTGATCCACATCTG |
| CD206 | GTGGGGACCTGGCAAGTATC | CACTGGGGTTCCATCACTCC |
| Arg-1 | ACATTGGCTTGCGAGACGTA | ATCACCTTGCCAATCCCCAG |
| VEGF-A | TGGGAGAACCCAAATGCTCC | CACTAGGCAACAGCACCTCA |
| TGF-β | ACTGGAGTTGTACGGCAGTG | GGGGCTGATCCCGTTGATTT |
| IL-10 | CCAAGGTGTCTACAAGGCCA | GCTCTGTCTAGGTCCTGGAGT |
| TNF-α | CCCTCACACTCACAAACCAC | ACAAGGTACAACCCATCGGC |
| CD86 | CTTACGGAAGCACCCACGAT | CGGCAGATATGCAGTCCCAT |
